# GlucoSense: early diabetes risk assessment via fusion of human activity recognition and glucose prediction

**DOI:** 10.3389/fdgth.2026.1932366

**Published:** 2026-10-14

**Authors:** Md Fokrul Islam Khan, Md Zia Uddin, Mohammed Shamsul Alam, Adel Baselizadeh, Abdul Kadar Muhammad Masum

**Affiliations:** 1 College of Business, Westcliff University, Los Angeles, CA, United States; 2 Sustainable Communication Technologies, SINTEF Digital, Oslo, Norway; 3 Department of Computer Science and Engineering, International Islamic University Chittagong, Chittagong, Bangladesh; 4 RITMO Centre for Interdisciplinary Studies in Rhythm, Time and Motion (IFI), Department of Informatics, Faculty of Mathematics and Natural Sciences, University of Oslo, Oslo, Norway; 5 Department of Computer Science and Engineering, Southeast University, Dhaka, Bangladesh

**Keywords:** continuous glucose monitoring, early diabetes diagnosis, graph attention network, human activity recognition, NIDDM, type 2 diabetes

## Abstract

Non-Insulin-Dependent Diabetes Mellitus (NIDDM) is a chronic and progressive metabolic disease characterized by chronically high blood glucose levels. It is a disease with an often asymptomatic onset, which means that a high percentage of affected individuals remain unidentified until quite far in the disease process, thereby underlining the importance of a good, reliable screening instrument for proactive detection. In this paper, GlucoSense is introduced as a single framework for early NIDDM risk stratification by combining Human Activity Recognition (HAR) with continuous blood glucose forecasting using two Graph Attention Network version 2 (GATv2)-based architectures. The first is a HAR module trained with smartphone triaxial accelerometer data that covers 23 activity classes, while the second is a glucose prediction module trained with the OhioT1DM dataset to predict glucose levels for a 30 min horizon. Unlike sequential models, GlucoSense encodes complex dependencies between movement patterns, cardiac activity, nutrition, and glycemic behaviour by modeling relationships in a graph structure. The study was longitudinal, spanning 90 days, with six participants. Three important clinical parameters, mean glucose level, time above hyperglycemic level, and glycemic coefficient of variance were calculated. The system was able to correctly identify metabolically healthy individuals and those with prediabetic patterns. The framework was cross-validated with HbA1c laboratory tests to verify the clinical consistency of the framework. Both modules were benchmarked against sequential, convolutional, attention-based, and graph-based baselines under an identical protocol with five independent runs. The glucose module lowered the root mean squared error from 31.82 mg/dL for an LSTM baseline to 25.60 mg/dL, and the activity module reached 98.50% accuracy, with every difference confirmed by paired significance testing and reported with 95% confidence intervals. Understanding glucose dynamics based on smartphone sensor data and a limited number of physiological markers, GlucoSense offers a non-invasive, scalable and cost-effective alternative to traditional clinical glucose screening. This paper demonstrates the feasibility of smartphone-based longitudinal diabetes risk assessment and motivates future large-scale population studies.

## 1 Introduction

Diabetes mellitus is one of the most important public health problems of today, both in terms of prevalence and incidence (1). In 2021, there were more than 500 million adults affected in the world, representing 10.5% of the adult population, and this is expected to rise to 12.2% by 2045. Type 2 Diabetes (also known as Non-Insulin-Dependent Diabetes Mellitus or NIDDM) makes up over 90% of all diagnosed diabetes. One aspect of this crisis that is particularly disturbing is that only about 45% of people diagnosed with a disease have a confirmed diagnosis, and the majority of these are NIDDM. The reason for this is mostly because of the fact that the disease has a way of developing without being noticed in its early stages and only causes an actual concern in later stages. If not diagnosed early, the condition progresses continuously, and when it is detected, management can become effective, but may not start soon enough, as noted by the study (2, 3). Together, these facts highlight the critical need for innovative, easy-to-access, and non-invasive screening strategies for NIDDM that do not rely on clinical manifestations.

Several clinical studies have shown that there is a strong relationship between lifestyle level of physical inactivity and increased susceptibility to NIDDM. Extended sitting, static standing and recumbency (sleeping) are all types of prolonged sitting, which have consistently been identified as a risk factor for poor glycemic outcomes, while regular exercise has been a consistent protective metabolic factor (4–7). Human Activity Recognition (HAR) is a computational paradigm for inferring and/or classifying physical behavioural states from raw sensor signals. It offers an intriguing and non-obtrusive way to continuously collect diabetes-relevant lifestyle indicators. Smartphones are ubiquitous and equipped with accelerometric sensors which make them a practical and privacy-friendly means to continuously monitor a user’s activity and health without requiring a camera or radiation.

Continuous Glucose Monitoring (CGM) has proven to be a valuable tool for measuring the temporal changes of blood glucose, both in research and clinical practice. CGM recordings provide high-level details of glycemic trajectories, which reflect a combination of food intake, activity, emotional stress, and intrinsic physiology. The predictive power of both classical machine learning and modern deep learning approaches to CGM data have been demonstrated, the majority of which are focused on Type 1 Diabetes Mellitus (T1DM) management. In spite of this progress, the current pipelines for CGM-based prediction have been developed and evaluated without considering them as part of more comprehensive behavioural monitoring systems. The known link between physical activity, dietary patterns, and glucose response has yet to be incorporated into a combined physical activity (HAR) and CGM prediction system for the detection of early NIDDM risk in early stages.

This present work aims to address this gap. We envision GlucoSense, a dual-module computational framework that integrates our GATv2-based classifier Human Activity Recognition (GATv2-GraphSensor) with a standalone GATv2-based model for continuous blood glucose forecasting, which are based on known physiological relationships between activity behaviour, dietary intake, glycemic regulation, and NIDDM risk. The glucose prediction module uses the publicly available OhioT1DM dataset (8), consisting of 8 weeks of multimodal recordings from patients with T1DM, to train a standalone GATv2 model predicting blood glucose 30 min in advance. The activity recognition module trains the GATv2-GraphSensor classifier with triaxial smartphone accelerometer data collected from twelve volunteers across 23 diabetes related activities. The frequency of sensor acquisition was set at 1 Hz, which was not dictated by recognition requirements but rather by a trade-off between recognition fidelity and power usage, as opposed to the 50 Hz frequencies used in previous studies (9, 10). Both modules were chosen to be implemented with GATv2 (11) due to its dynamic attention formulation, which jointly encodes relevance scores for both the source and target node representations, resulting in richer relational encodings than the static scoring of the original GAT (12). This directly helps bridge the previously mentioned problems of LSTM, CNN and DBN model which have been successful in HAR and CGM forecasting tasks.

To validate this, heart rate, accelerometer and dietary data were collected from six participants over a 90-day period with a custom-built Android application and a Fitbit Charge device. The pre-trained GATv2-GraphSensor model was used to classify accelerometer recordings and the activity labels were combined with cardiac and dietary input to create a comprehensive multimodal feature set. The consolidated representation was then used as the input to the stand-alone GATv2 model to create individual 90-day blood glucose trajectories. The predicted profiles were compared with reference glycemic thresholds and resulted in a diabetes risk classification for each participant. All 6 subjects were then formally tested in a laboratory and the clinical results agreed with the classification provided by the system, which offers preliminary support for the proposed method within this small proof of concept cohort.

Through prediction of risk, GlucoSense helps to direct people to clinical consultation and to decrease their reliance on expensive planned visits for examination, as well as the need for symptom presentation to get medical care. The framework, which relies on a smartphone, a heart rate monitor and self-entered meal logs, is accessible to the community in a practical, economically viable and non-invasive fashion to identify early cases of NIDDM.

The principal contributions of this work are as follows:

1. A novel framework, *GlucoSense*, is proposed for early NIDDM risk assessment by integrating smartphone-based Human Activity Recognition and Continuous Glucose Monitoring-based blood glucose prediction through two GATv2-based graph learning models.
2. A new human activity recognition dataset was developed from triaxial smartphone accelerometer signals collected across 23 diabetes-related activities under four smartphone orientations and both indoor and outdoor environments.
3. A clinically grounded risk stratification framework was developed using CGM-derived glycemic indicators, including mean glucose concentration, time above hyperglycemic thresholds, and coefficient of variation.
4. The proposed framework was validated on six experimental subjects using 90-day activity, meal, and heart rate records. The generated risk classifications were checked with independent HbA1c laboratory assessments.

The remainder of this article is organized as follows. Section 2 presents a review of the pertinent related literature. Section 3 describes the datasets employed in this study. Section 4 details the proposed methodology in full. Section 5 presents and discusses the experimental results, and Section 6 concludes the paper.

## 2 Literature review

A strong association of physical inactivity and the onset of NIDDM has been well established by extensive epidemiological investigation. When movement across behaviours (e.g., long periods of sitting in a chair, sitting without moving, prolonged recumbency) was examined, it was found to increase glycemic risk (4, 5). Regular exercise that can be sustained as a form of treatment will provide a meaningful protective effect from metabolic decline; higher intensity of exercise will provide a higher protective effect. Colberg et al. (6) established the ability of regular and periodic exercise to reduce the elevation of postprandial glucose and decrease the very low-density lipoprotein triglyceride (VLDL-TG) in the circulation. A 12 year prospective cohort study of 87,253 American women ages 34 to 59 identified a 2/3 reduction in risk of developing NIDDM among those in the most active activity category compared to the least active category (7). These observations further highlight the clinical rationale of combining a HAR module in a framework that addresses detection of early NIDDM risk.

Research in activity recognition began in the 1980s and has continued in various areas including clinical monitoring, sports analytics, robotics, assistive technology, and older adults care (13). Various types of sensors have been studied to classify human movement, such as accelerometric sensors, gyroscopic sensors, thermal sensors, and optical sensors (14–18). While vision-based recognition systems, like the ones that have been implemented in the past and have done well (19–22) have limitations, they are useful in raising important questions about privacy. Technical modalities such as wearable and radio-frequency based modalities are effective but come with radiation exposure concerns and are geographically or infrastructurally limited (23–27). Smartphone-based HAR occupies a useful middle ground between the ubiquity of the devices and the lack of health and privacy concerns of other sensing paradigms, such as those mentioned in (28–30). To validate the potential of inertial sensors embedded in smartphones for health-related HAR, Guidoux et al. (31) built an energy expenditure estimation system based on activities. Later work has shown that these single axis accelerometer measurements can accurately classify the motion with satisfactory accuracy (32–34). One of the drawbacks in the previous HAR systems is that they were operating with only 50 Hz data acquisition, which consumes a huge amount of energy from mobile devices (9, 10). In the present work, an attempt is made to deliberately run the system at 1 Hz so that a prolonged continuous monitoring can be carried out with limited battery consumption.

Numerous deep learning models have been used for smartphone HAR using accelerometer data. Ignatov et al. (33) designed an activity classifier with 93.32% accuracy on WISDM and 97.62% on UCI dataset by using a convolutional classifier for six activity classes. Using a Deep Belief Network, Alsheikh et al. (35) obtained results of 89.38%, 91.5% and 98.23% on the Skoda, Daphnet and WISDM corpora, respectively, when compared to the results of traditional classifiers. Ha and Choi (32) proposed a multimodal architecture based on 2-D convolutional filters, achieving 98.26% and 97.92% on M-Health and Skoda, respectively. Xia et al. (36) made a hybrid LSTM-CNN based design with accuracy of 95.78%, 95.85%, and 92.63% on UCI-HAR, WISDM and OPPORTUNITY respectively. Uddin and Soylu (27) proposed a Human action Recognition model using LSTM which achieved 99% accuracy on the MHEALTH benchmark with wearable sensors data. Bao and Intille (37) recorded the body signals from biaxial accelerometers from five body positions and concluded that the signals measured by the accelerometers mounted on the thighs were the most informative, and they were able to reach an accuracy of more than 80% by using a Decision Tree. All of these architectures have their own drawbacks. Vanishing and exploding gradients are still a problem in LSTM networks, which makes them susceptible to fail when learning to model long-range dependencies. CNNs have been designed for inputs that are spatially regular, and hence do not readily adapt to non-uniform temporal physiological signals. One of the major challenges of the DBNs is their high initialization overhead that makes their practical deployment on resource constrained mobile platforms difficult. Graph Attention Networks overcome these constraints by operating directly on graph-structured inputs, by efficiently processing nodes in parallel, and by weighting the importance of neighbouring data based on data without requiring a graph topology specification (12). This paper builds upon these benefits, by applying a variant of the original attention mechanism, GATv2 (11) to overcome the limitation of static attention in the original formulation, by conditioning relevance scores on source and target node representations together. This yields only more expressive relational encodings, and the complicated interdependencies that occur in physiological sensor data renders GATv2 especially suitable.

In multiple clinical areas, HAR has proven to be a useful tool. Activity monitoring was used to describe behavioural signatures in schizophrenia patients by Umbricht et al. (2020) (38). Islam et al. (39) proposed a hierarchical feature fusion strategy for multimodal HAR in digital health applications. Papadopoulos et al. (40) used inertial sensors to aid in the screening for Parkinson’s disease. Preece et al. (41) showed that the recognition of movement profiles may be used to uncover links between activity profile and neurological disorders. In spite of this variety of uses, HAR has been paid little attention as part of NIDDM specific early risk assessment pipelines.

Work published from 2024 onwards has moved sensor based activity recognition towards label efficient and relation aware modelling, and this recent literature is summarised here to place the present contribution in an up to date context. Yuan et al. (42) pre-trained an accelerometer encoder on approximately 700,000 person days of unlabelled UK Biobank recordings and reported consistent improvements over strong supervised baselines across eight downstream activity recognition datasets, with the learned representations transferring across external cohorts, living environments and sensor devices. Haresamudram et al. (43) investigated discrete representations learned through self-supervision for wearable activity recognition, forming part of a broader movement away from fully supervised training. Leng et al. (44) exploited language driven cross modality transfer to synthesise inertial training data, which relaxes the annotation burden that constrains the introduction of new activity taxonomies. On the architectural side, Navakauskas and Dumpis (45) systematically compared dynamic recurrent architectures for wearable activity recognition and characterised the trade off between classification accuracy, computational cost and interpretability, a trade off that is central to continuous smartphone deployment. Bernardo et al. (46) addressed the position dependence of smartphone datasets and proposed a position independent recognition system trained on signals captured with the handset carried at different body locations, a concern that directly motivated the four orientation acquisition protocol adopted in the present study. Graph based formulations have gained parallel traction. Mondal et al. (47) argued that convolutional pipelines learn from each smartphone sensor sample in isolation and therefore discard the structural information that links samples to one another, and demonstrated that an end to end graph neural network recovers this information while reducing the training data requirement. The GATv2-GraphSensor module proposed in the present work belongs to this relational family and extends it by replacing the static attention of the original graph attention formulation with dynamic attention.

CGM has evolved into a useful research and clinical tool for studying glycemic dynamics at the micro level. Zeevi et al. (48) compiled CGM and dietary data for 800 people and used a machine learning approach to personalize postprandial glycemic response predictions, which were then validated using a controlled trial. Tily et al. (49) also showed that adding the gut microbiome profile to nutritional and anthropometric data further enhanced the fidelity of glycemic prediction. Glucose forecasting using CGM has been extensively used for deep learning. Mohebbi et al. (50) compared logistic regression, CNN and MLP variants with a deep learning algorithm for detecting insulin adherence in T2DM with simulated CGM signals. Wang et al. (51) employed an AdaBoost regression model with glucose curves derived from CGM data to classify diabetes subtypes with an overall clinical coincidence rate of 90.3%. A multi-layer Convolutional Recurrent Neural Network (CNN) trained using glucose, carbohydrate, and insulin signals achieved RMSEs of 21.07 and 33.27 mg/dL at 30- and 60 min time horizons respectively (52). In the T1DM setting, an analogous CRNN achieved 11.22 and 17.45 mg/dL MAE at 30 min and 23.25 and 33.67 mg/dL at 60 min (53). To mitigate the impact of measurement noise in CGM data, Rabby et al. (54) designed an LSTM-based recurrent model with Kalman-smoothed CGM signals and insulin dose, carbohydrate and step count features.

Physical activity has been a consistent interesting predictor variable in the field of glycemic modelling research. A study which integrated activity and stress signals with CGM data found that adding activity data reduced the 1 h glucose prediction MAE from 35.1 to 31.9 mg/dL, and the addition of stress further decreased the MAE to 14.2 mg/dL (55). De Paoli et al. (56) compared several learning techniques for blood glucose forecasting in T1DM subjects during physical activity and reported that the choice of learning algorithm has a marked effect on accuracy once exercise is present in the input. The aerobic and anaerobic protocols were evaluated in a Jump Neural Network for rapid glucose changes due to exercise in T1DM subjects, with RMSEs of 26.3 and 20.8 mg/dL respectively.

There are several modelling paradigms for hypoglycemia prediction using CGM. Nocturnal hypoglycemia sensitivity was better than 94% in both the prediction window of 30 and 60 min, with a support vector regression approach (57). Woldaregay et al. (58) proposed a one-class classifier for detecting hyperglycemia induced by infection in T1DM, outperforming density and reconstruction based classifiers by the ones proposed by them in their paper.

Predicting nocturnal hypoglycemia by comparative modelling studies has yielded helpful performance measures. In T1DM patients admitted to the hospital, Berikov et al. (59) found that the random forest had the highest AUC of 0.97 and 0.942 at 15 and 30 min after the model was trained by lasso logistic regression and neural network. Jensen et al. (60) used a linear discriminant analysis model for the analysis of 32 multi-source features extracted from CGM, insulin, meal and demographic data, yielding 0.79 AUC with 70% specificity and 75% sensitivity. A SVM and MLP model were tested by Bertachi et al. (61) for nocturnal hypoglycemia prediction, and SVM was found to be best at the population level, with 82.15% specificity and 78.75% sensitivity.

Other contributions have focused on personalization and uncertainty quantification of forecasting. In T1DM, T2DM and gestational diabetes, Seo et al. (62) identified the CNN to be predictive at 15, 30, 45 and 60 min with the glucose at the same time as one of the most powerful predictors. Martinez et al. (63) embedded pharmacokinetic insulin and carbohydrate absorption curves into an RNN-LSTM model and achieved the error in predicting the insulin level was around 9.2 mg/dL. Zhu et al. (64) proposed a fast-adapting attention recurrent model with meta-learning for rapid personalized patient-level personalization. Kalita et al. (65) presented stacked LSTM-GRU-LSTM network which achieved RMSE of 5.27 mg/dL after 15 min and RMSE of 14.85 mg/dL after 30 min. Using evidential regression for uncertainty quantification (UQ), an IoMT optimized lightweight recurrent model performed better than ARIMA, TCN, CRNN, LSTM, Bi-LSTM, and SVR in terms of prediction error and hypoglycemia detection, as shown in (66).

Clarke Error Grid analysis has served as a typical clinical validation tool in glucose prediction studies. Pappada et al. (67) reported that a feed-forward network for extended glucose forecasting placed over 99% of their outputs in safe CEG zones. Herrero et al. (68) demonstrated the accuracy of the SVM-based classification on glycemic features for the identification of CGM contributor was 86.8%. Thyde et al. (69) achieved an insulin adherence classification accuracy of 79.8% on the mean basis with a CNN model that included engineered and learned features. Cappon et al. (70) used XGBoost for classification of postprandial glycemic status with an AUC of 0.84. Marling et al. (71) built a Consensus Perceived Glycemic Variability metric using SVR and multilayer perceptrons, with an overall accuracy of 90.1% and specificity of 74.1%.

Research published from 2023 onwards has been dominated by attention based, graph based and pre-trained architectures, and this recent body of work is reviewed here so that the literature reflects the current state of the field. Piao et al. (72) proposed graph attentive recurrent neural networks that organise multivariate CGM streams as a graph and use graph attention to rank the contribution of each physiological variable, achieving the best accuracy among fifteen established baselines across four datasets that span both type 1 and type 2 diabetes while additionally producing interpretable feature maps for postprandial excursions. Li et al. (73) represented multivariate glucose data as a hypergraph and dynamically partitioned it into subgraphs to separate strongly and weakly correlated variables, outperforming seventeen transformer based and graph based forecasting baselines. Within the sequence modelling family, Xiong et al. (74) placed a transformer encoder and a long short term memory branch in parallel so that global and local temporal dependencies are captured simultaneously, while Zhu et al. (75) showed that a transformer forecaster can be specialised to distinct patient populations and executed on edge hardware. Maqsood et al. (76) combined attention based multi task learning with a decision transformer to deliver personalised and explainable glucose forecasts. A further shift is the emergence of pre-trained sensor foundation models. Luo et al. (77) pre-trained a transformer decoder on 1.6 million CGM records contributed by patients of different diabetes types, ages and genders, reporting a 48.51% reduction in root mean squared error at the one hour horizon together with stable zero shot performance on held out patient groups. These developments motivated the dynamic graph attention formulation adopted in the present work and shaped the baseline set used for the comparative experiments reported in Section 5.

Though progress has been made in both HAR and CGM-based glycemic prediction, there has been no previous effort to combine the two in a single model specifically for the purpose of early identification of NIDDM. This represents a significant methodological lacuna since the physiological interactions between movement behaviour, food consumption and glycaemic control are highly interdependent and collectively influence the development of NIDDM. Existing clinical diagnostic testing strategies for NIDDM, such as fasting plasma glucose testing, oral glucose tolerance testing, and glycated hemoglobin (HbA1c) testing are time consuming, invasive, and used only after clinical signs of the disease have been observed, thereby missing the window for optimal and effective early intervention.

The GlucoSense framework that is proposed in this paper fills this gap by combining a GATv2-based HAR classifier trained on smartphone accelerometer signals with a GATv2-based blood glucose prediction model trained on the OhioT1DM dataset (8). The HAR module is used to predict the activity labels, which are then correlated with heart rate and nutritional intake information and sent to the CGM model for 30 min prediction that yielded an average RMSE of 25.60 mg/dL and 94.30% Zone A on the Clarke Error Grid. These resulting glycemic profiles are then compared to clinical thresholds to create a non-invasive and cost-effective early risk categorization. It only needs a smartphone, a cardiac monitor and the manual recording of dietary data, and early identification of the risk of NIDDM is put into action before symptoms set in.

## 3 Datasets description

### 3.1 Dataset for continuous glucose monitoring

The OhioT1DM dataset (8) provided the primary source for blood glucose level prediction in this work. The collection contains eight weeks of longitudinal multimodal data from 12 individuals with T1DM, all maintained on insulin pump therapy with concurrent CGM. Each participant used a Medtronic 530G or 630G pump paired with a Medtronic Enlite sensor throughout the observation window. The dataset was published in two sequential cohorts. The first cohort, released in 2018, encompasses six participants (IDs: 559, 563, 570, 575, 588, 591) who wore Basis Peak Fitness trackers. The second cohort, released in 2020, includes six further participants (IDs: 540, 544, 552, 567, 584, 596) equipped with Empatica Embrace devices. Together, the 12 individuals represent a range of age groups and both sexes, as depicted in Figure 1.

**Figure 1:**
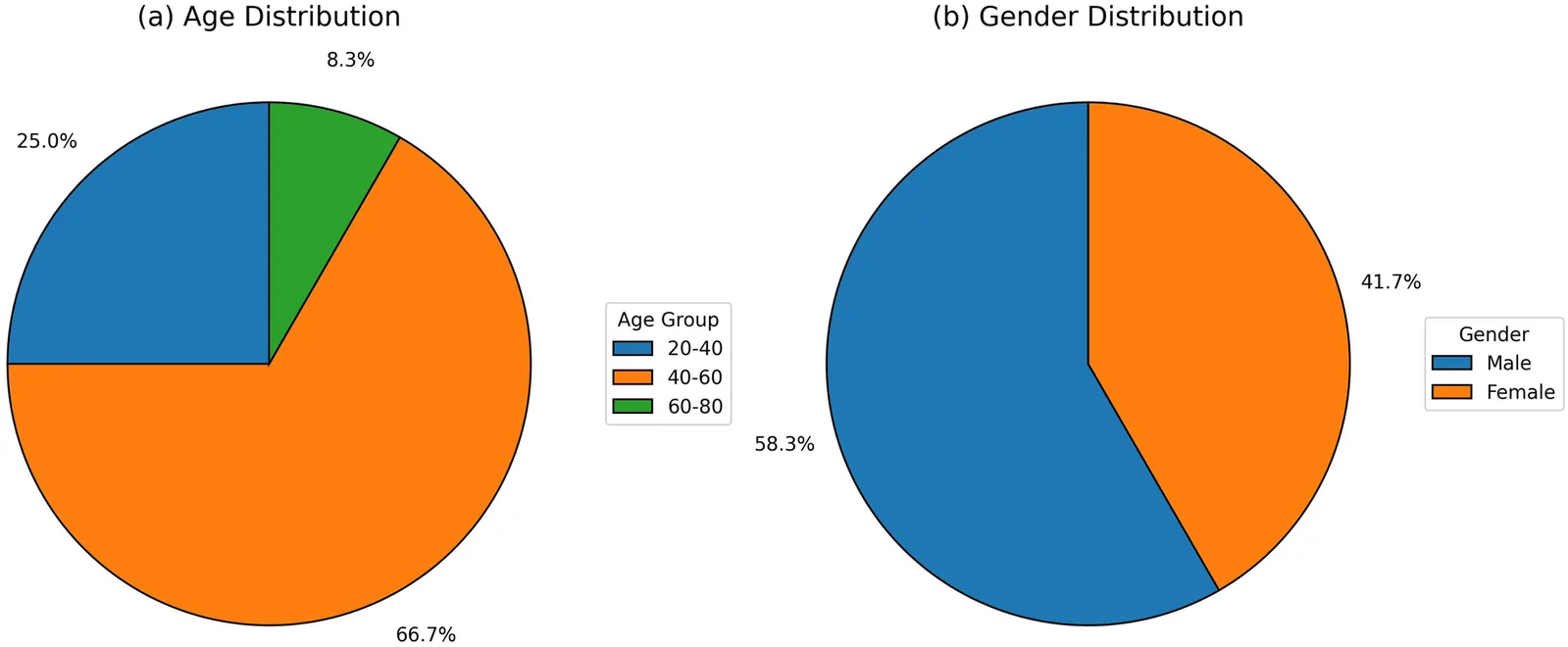
Demographic composition of the twelve OhioT1DM participants. (a) Age distribution. (b) Gender distribution.

Per-participant recordings include five-minute CGM glucose readings, finger-prick self-monitoring measurements, basal and bolus insulin administration logs, self-reported meal times with carbohydrate estimates, and annotated life events covering exercise, sleep, work, stress, and illness. Physiological wearable data from Basis Peak devices include five-minute aggregated heart rate, galvanic skin response, skin temperature, ambient temperature, and step counts. Empatica Embrace recordings provide one-minute aggregations of galvanic skin response, skin temperature, and movement magnitude. Table 1 lists all available variables. Representative CGM profiles from the final seven days and the complete test sequences of six participants are displayed in Figure 2. Features selected for this study were: Patient, Glucose Level, Finger Stick, Meal, Sleep, Work, Stressors, Hypo Event, Exercise, Basis Heart Rate, and Acceleration.

**Table 1:** Summary of variables in the OhioT1DM dataset.

Variable	Description
Patient	Patient ID and insulin type
Glucose Level	Continuous glucose monitoring (CGM) data recorded every 5 min
Finger Stick	Self-monitored blood glucose measurements
Basal	Continuous basal insulin infusion rate starting at timestamp $t_{s}$
Temp Basal	Temporary basal rate overriding normal basal; 0 indicates suspension
Bolus	Insulin dose (normal or extended) administered for meals or hyperglycemia
Meal	Meal time, type, and estimated carbohydrate intake
Sleep	Sleep periods with subjective quality (1 = Poor, 2 = Fair, 3 = Good)
Work	Work time intervals with intensity (1–10 scale)
Stressors	Time of self-reported stress events
Hypo Event	Time of hypoglycemic episodes (symptoms not included)
Illness	Time of self-reported illness
Exercise	Exercise duration and intensity (1–10 scale)
Basis Heart Rate	Heart rate aggregated every 5 min (Basis Peak users only)
Basis GSR	Galvanic skin response; 5 min (Basis) or 1 min (Empatica) aggregation
Skin Temperature	Skin temperature (${}^{\circ }$F); 5 min (Basis) or 1 min (Empatica)
Air Temperature	Air temperature (${}^{\circ }$F), 5 min aggregation (Basis only)
Steps	Step count aggregated every 5 min (Basis only)
Basis Sleep	Sensor-detected sleep periods with optional quality score
Acceleration	Acceleration magnitude aggregated every 1 min (Empatica only)

**Figure 2:**
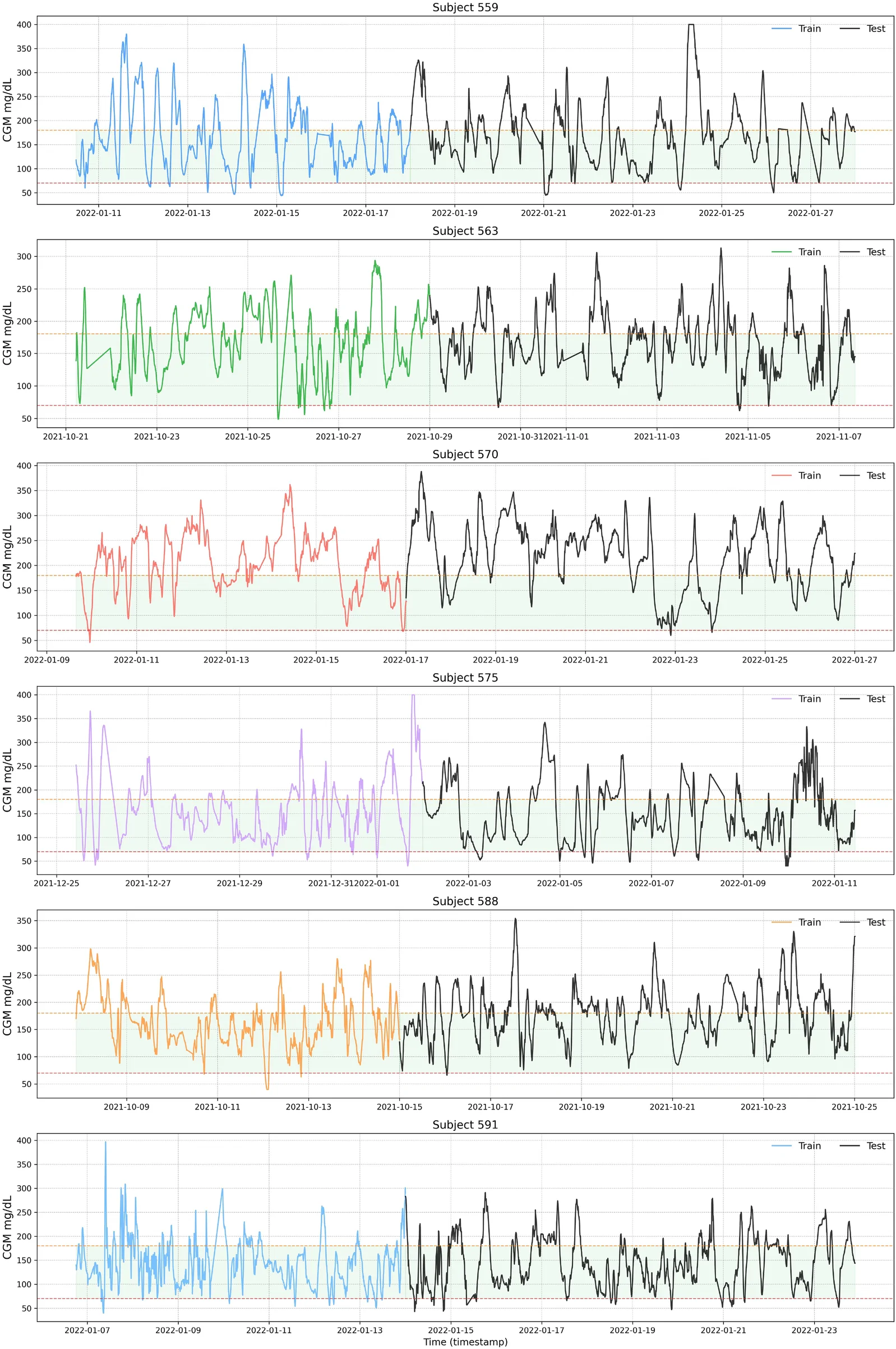
Representative CGM recordings from the final seven days of the training window and a complete test sequences for six OhioT1DM participants.

### 3.2 Sensor data and targeted activities

Inertial sensing via smartphone accelerometers has produced strong HAR results in prior literature (33, 35, 37), and this study follows that established direction. A purpose-built smartphone application was used to capture triaxial accelerometer readings from 23 activity categories at a 1 Hz acquisition rate. A triaxial accelerometer quantifies linear acceleration, displacement, and velocity along orthogonal x, y, and z axes simultaneously.

The 23 activities were selected for their established associations with NIDDM-related physiological symptoms: Walking-normal, Walking-moderate, Walking-vigorous, Standing, Walking upstairs-normal, Walking upstairs-moderate, Walking upstairs-vigorous, Walking Downstairs-normal, Walking Downstairs-moderate, Walking Downstairs-vigorous, Drinking, Eating, Itching, Lying, Using toilet, Jogging, Cycling, Irrelevant activities, Driving, Sitting, Exercise-dips, Exercise-leg raise, and Push up. A 200 s sample of signals from four representative activities is shown in Figure 3.

**Figure 3:**
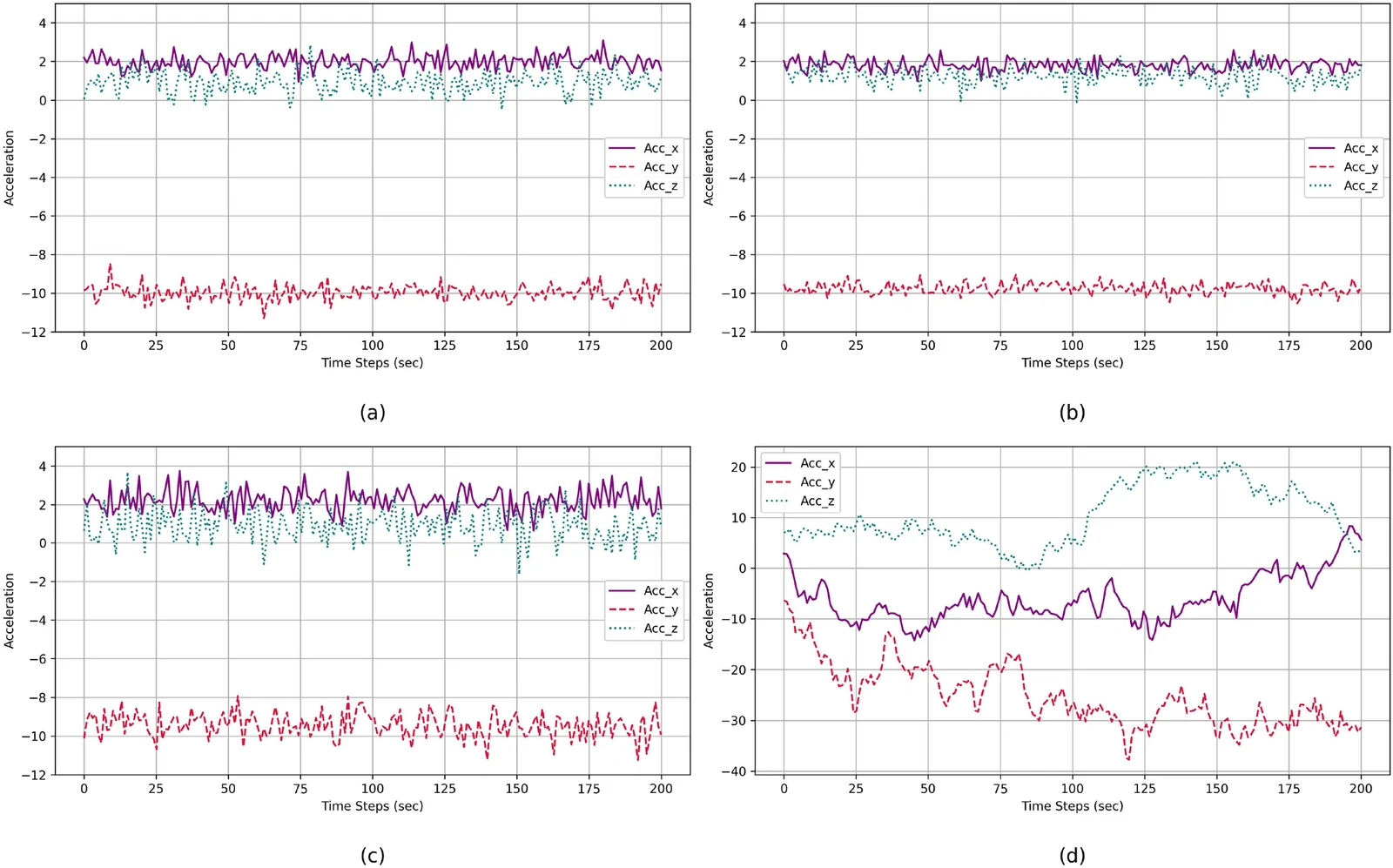
Triaxial accelerometer signals over the first 200 s for four representative activities: **(a)** sitting, **(b)** standing, **(c)** driving, **(d)** jogging.

Cardiovascular activities, including walking, jogging, and cycling, as well as resistance-based movements such as push-ups and leg raises, have been associated with blood glucose variability and metabolic health outcomes. Eating, drinking, and toilet use were included to capture behavioural markers linked to carbohydrate intake, hydration, and urinary frequency. Activities such as lying, sitting, scratching, and standing provide indicators of physical inactivity and type 2 diabetes-associated discomfort patterns. To enable finer-grained glycemic risk stratification, movement-based activities were divided into normal, moderate, and vigorous tiers reflecting proportional differences in energy expenditure.

Twelve volunteers participated in data collection, equally divided between younger participants and those aged 50 and above. All were in good general health with no recorded medical conditions. Approximately 7,500 samples were gathered per diabetic-relevant activity, while irrelevant activity recordings totalling around 36,000 instances were retained separately to reduce misclassification rates. Irrelevant recordings captured phone use during sitting, standing, or reclined positions, as well as brief periods when the device was placed on a surface. To address variability in how individuals carry their smartphones, data were collected across four pocket orientations including upright, inverted, and flipped positions. Recordings spanned both indoor and outdoor settings, with walking data gathered in shopping centres, residential streets, and indoor corridors. Seated behaviours were recorded in both standard chair positions and squatting postures, including use of both elevated and standard toilet fixtures. The complete dataset totals 201,000 instances across 23 activity classes.

## 4 Proposed system

This section describes the construction and execution of the proposed framework. Figure 4a illustrates the complete sequence of steps followed throughout the system pipeline.

**Figure 4:**
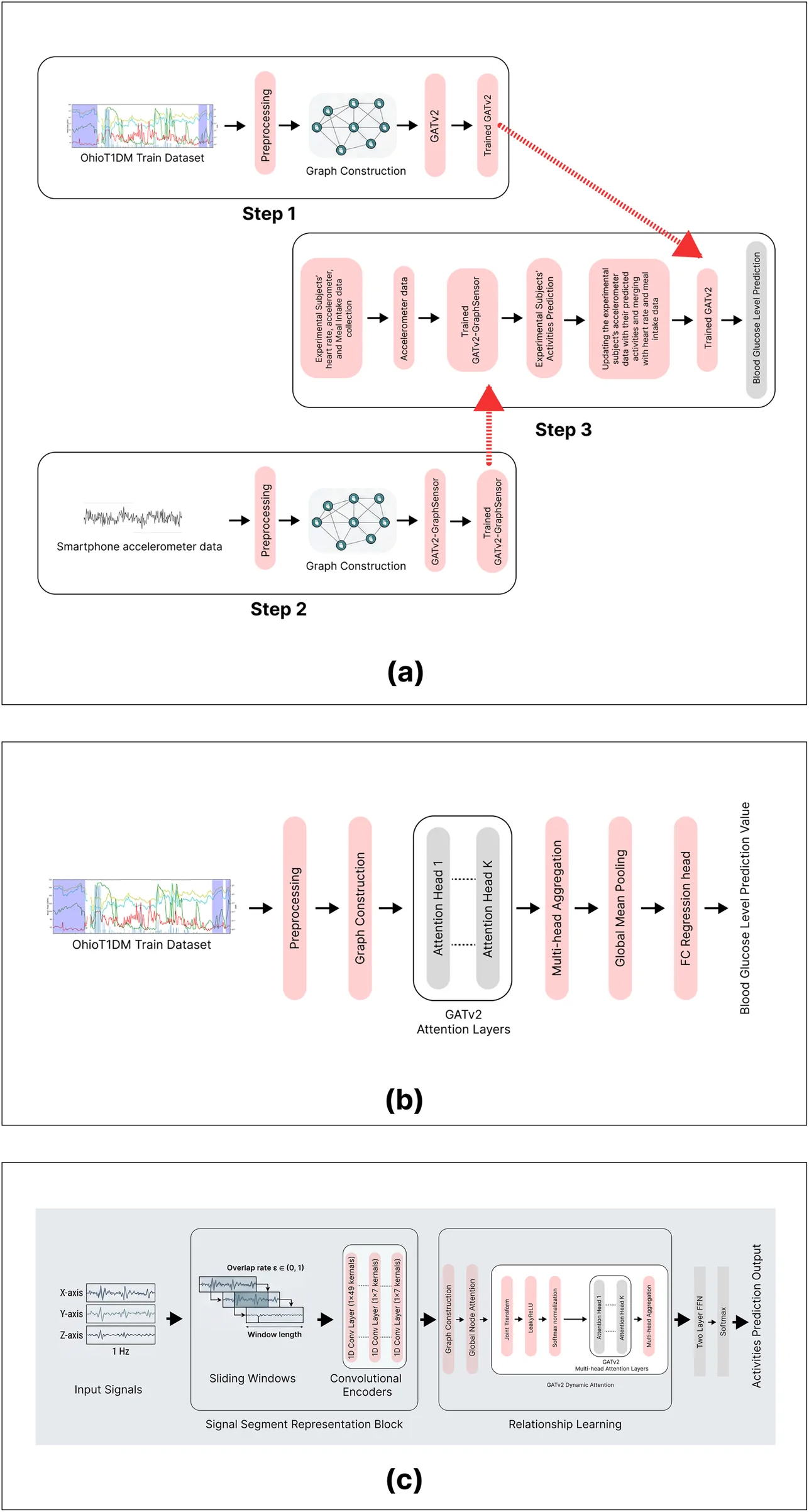
**(a)** End-to-end system pipeline for 30 min blood glucose level prediction via fused HAR and CGM modelling. **(b)** Standalone GATv2 model architecture for blood glucose level prediction from OhioT1DM physiological feature graphs. **(c)** GATv2-GraphSensor pipeline for smartphone accelerometer-based Human Activity Recognition, showing the signal segment representation and relationship learning blocks.

### 4.1 Overall architecture description

As shown in Figure 4a, the system operates across three consecutive stages. In Step 1, a standalone GATv2 model for blood glucose level prediction was trained on the OhioT1DM dataset. Prior to graph construction, the raw multimodal data underwent preprocessing before being organized into a graph structure G=(N,L), where each node represented a temporal feature instance and each edge encoded the relationship between pairs of nodes. The GATv2 model was trained on this graph to capture complex inter-feature dependencies governing glycemic dynamics.

In Step 2, a GATv2-GraphSensor model for Human Activity Recognition was trained on smartphone accelerometer data. The GATv2-GraphSensor architecture was adapted from the GraphSensor model introduced by Ge et al. (78), which demonstrated strong performance on sensor-based activity recognition tasks, and extends it by replacing the original GAT attention mechanism with the GATv2 formulation. Raw sensor signals were preprocessed and subsequently converted into a graph representation following an analogous construction process. The trained GATv2-GraphSensor model learned to identify physical activity patterns known to influence glycemic response.

Step 3 constitutes the inference pipeline applied to the experimental subjects. Heart rate, accelerometer, and meal intake data were collected from each participant. The accelerometer signals were processed by the pre-trained GATv2-GraphSensor model to generate activity class predictions. These predicted activity labels were consolidated with the corresponding heart rate and meal intake records to form a unified multimodal feature representation. This integrated feature set was subsequently supplied to the pre-trained GATv2 model to produce subject-specific blood glucose level predictions at a 30 min horizon. Each component of the architecture is described in detail in the subsections that follow.

### 4.2 Step 1: blood glucose level prediction

A GATv2 architecture, as illustrated in Figure 4b, was constructed to predict blood glucose levels by jointly modelling physiological, behavioural, and dietary signals derived from the OhioT1DM dataset. GATv2 (11) was adopted in place of the original GAT formulation to address its well-documented limitation of static attention, wherein attention scores are computed independently of the query node’s representation. GATv2 introduces a dynamic attention mechanism that conditions the attention score jointly on both the source and target node representations, yielding strictly more expressive attention functions.

#### 4.2.1 OhioT1DM data preprocessing

The following features were extracted from the OhioT1DM dataset for blood glucose prediction: glucose level from CGM, finger stick readings, meal intake with carbohydrate estimates, sleep quality, work intensity, stress events, hypoglycemic episodes, exercise duration and intensity, heart rate, and acceleration magnitude. These multimodal inputs collectively captured the physiological and behavioural factors governing glycemic dynamics.

Prior to graph construction, all extracted features were preprocessed to ensure consistency and data quality. Missing CGM readings arising from sensor dropout were imputed using linear interpolation to preserve temporal continuity. All numerical features were subsequently standardized using z-score normalization, defined as in Equation 1:

$${\tilde{z}}_{i}=\frac{z_{i}-\mu_{z}}{\sigma_{z}}$$ (1)

where $z_{i}$ denotes the raw value of feature z, $\mu_{z}$ and $\sigma_{z}$ are its mean and standard deviation respectively, and ${\tilde{z}}_{i}$ is the resulting normalized value. Since CGM sensors recorded readings at five-minute intervals, all remaining features were aligned to this temporal resolution through forward-filling, ensuring a consistent time axis across all input modalities.

#### 4.2.2 Graph construction

The preprocessed features were organized into a graph G=(N,L) to explicitly represent interdependencies among heterogeneous physiological and behavioural signals. Each node n∈N represents a distinct feature stream at a given timestep, such as heart rate, acceleration magnitude, or meal carbohydrate content, and is initialized with its corresponding normalized feature vector. An edge $l_{ab}\in L$ is established between nodes $n_{a}$ and $n_{b}$ when the Pearson correlation coefficient between their temporal signals exceeds a predefined threshold ϕ, reflecting statistically meaningful co-variation between the two feature streams. This graph formulation enables the model to jointly capture the influence of physical activity, dietary intake, and physiological signals on glucose dynamics, rather than treating each modality in isolation.

#### 4.2.3 GATv2 architecture for blood glucose level prediction

The GATv2 model operates over the heterogeneous feature graph G=(N,L), where each node corresponds to a distinct physiological or lifestyle variable. The GATv2 attention mechanism (11) dynamically assigns importance weights to neighbouring nodes by jointly transforming both the source and target node representations before computing the attention score. This distinguishes GATv2 from the original GAT, in which the linear transformation is applied to each node independently prior to concatenation, resulting in attention scores that do not vary with the query node.

For each node $n_{a}\in N$ with feature vector $r_{a}\in R^{c}$, where c denotes the feature dimensionality, the GATv2 attention layer computes the unnormalized attention score $e_{ab}$ between nodes $n_{a}$ and $n_{b}$ as in Equation 2:

$$e_{ab}=q^{⊤}\;\text{LeakyReLU}\left(P_{s}\;r_{a}+P_{t}\;r_{b}+b\right)$$ (2)

where $P_{s}\in R^{c^{\prime }\times c}$ and $P_{t}\in R^{c^{\prime }\times c}$ are separate learnable linear transformation matrices applied to the source node $n_{a}$ and target node $n_{b}$ respectively. $b\in R^{c^{\prime }}$ is a learnable bias vector. $q\in R^{c^{\prime }}$ is a learnable attention vector. LeakyReLU is applied after the joint transformation, enabling the attention function to vary with both the source and target representations simultaneously. This is the key distinction from the original GAT formulation.

The normalized attention coefficient $\gamma_{ab}$ is obtained by applying Softmax over the neighbourhood $M_{a}$ of node $n_{a}$ (Equation 3):

$$\gamma_{ab}=\frac{\exp\;(e_{ab})}{\sum_{k\in M_{a}}\exp\;(e_{ak})}$$ (3)

where $M_{a}$ denotes the set of neighbouring nodes of $n_{a}$. The updated node representation $r_{a}^{\prime }$ is computed as in Equation 4:

$$r_{a}^{\prime }=\sigma \left(\sum_{b\in M_{a}}\gamma_{ab}\;P_{s}\;r_{b}\right)$$ (4)

where σ denotes the ELU activation function. Multi-head attention with K independent heads was employed for representational stability. For intermediate layers, head outputs were concatenated (Equation 5):

$$r_{a}^{\prime }={\|}_{k=1}^{K}\;\sigma \left(\sum_{b\in M_{a}}\gamma_{ab}^{k}\;P_{s}^{k}\;r_{b}\right)$$ (5)

where $\gamma_{ab}^{k}$ and $P_{s}^{k}$ denote the attention coefficient and source transformation matrix of head k. At the final attention layer, head outputs were averaged to yield a fixed-dimensional node embedding (Equation 6):

$$r_{a}^{\text{out}}=\sigma \left(\frac{1}{K}\sum_{k=1}^{K}\sum_{b\in M_{a}}\gamma_{ab}^{k}\;P_{s}^{k}\;r_{b}\right)$$ (6)

Global mean pooling over all node embeddings $\{r_{a}^{\text{out}}{\}}_{n_{a}\in N}$ then produced the graph-level representation, aggregating contributions from all physiological feature nodes into a single fixed-dimensional vector. This was passed through a fully connected regression head to generate the predicted blood glucose concentration at a 30 min horizon.

The GATv2 model was trained on OhioT1DM data from individuals with T1DM undergoing insulin therapy, while the experimental subjects were assessed for NIDDM. Although the two conditions differ pathophysiologically, the model does not depend on disease-specific labels. It learns generalized glucose dynamics from multivariate time-series signals, including those arising from physical exertion, nutritional intake, and physiological stress. Postprandial glucose excursions and exercise-driven fluctuations share temporal characteristics across patient populations (6, 55), meaning the learned representations remain informative for relative glycemic modelling across groups. This position is supported by published transfer studies. Deng et al. (79) reported that a single network family and transfer strategy performed well both on a type 2 diabetes cohort and on OhioT1DM, and Yu et al. (80) transferred glucose forecasters to previously unseen subjects with type 2 diabetes. Yang et al. (81) obtained the same result through clustering based domain adaptation, while Piao et al. (72) and Luo et al. (77) evaluated graph attention and pre-trained forecasters across cohorts containing both diabetes types. A fuller discussion of this evidence is given in Section 5.4. The OhioT1DM dataset’s multimodal richness, combining CGM, physiological signals, and annotated life events, makes it well-suited for training graph-based predictive models. Predicted glucose values are not treated as direct clinical measurements. Rather, they are interpreted through established glycemic indicators including mean concentration, time above hyperglycemic thresholds, and coefficient of variation. This risk-centred interpretation prioritizes relative glucose trajectory patterns over absolute accuracy, thereby limiting sensitivity to cross-population domain differences.

### 4.3 Step 2: human activity recognition

A GATv2 classifier inspired by the GraphSensor architecture (78) was employed to recognize physical activity patterns from smartphone accelerometer data. The original GraphSensor model was built upon the standard GAT formulation (12). In this work, the attention mechanism was upgraded to GATv2 (11) to overcome the static attention limitation inherent in the original GAT, wherein attention scores are computed independently of the querying node. GATv2 introduces a dynamic attention mechanism that jointly conditions the attention score on both the source and target node representations, producing strictly more expressive relational representations.

#### 4.3.1 Smartphone sensor data preprocessing

The smartphone accelerometer dataset was partitioned into 80% for training and 20% for testing. As no missing values were present, imputation was not required. Outlier detection was performed using the interquartile range method. A data point was classified as an outlier if it lay above the upper bound or below the lower bound defined in Equations 7, 8:

$$\text{Upper Bound}=Q_{3}+1.5\;(Q_{3}-Q_{1})$$ (7)

$$\text{Lower Bound}=Q_{1}-1.5\;(Q_{3}-Q_{1})$$ (8)

where $Q_{1}$ and $Q_{3}$ denote the first and third quartiles of the feature distribution, respectively. Detected outliers were substituted with the mean value of the corresponding axis rather than being removed, preserving the structural continuity of the time series.

All features were subsequently standardized using z-score normalization (Equation 9):

$${\tilde{a}}_{i}=\frac{a_{i}-\mu_{a}}{\sigma_{a}}$$ (9)

where $a_{i}$ denotes the raw sensor reading, $\mu_{a}$ and $\sigma_{a}$ are the mean and standard deviation of the corresponding feature respectively, and ${\tilde{a}}_{i}$ is the normalized output. A resultant magnitude feature was additionally derived from the three accelerometer axes as follows (Equation 10):

$$\rho =\sqrt{s_{x}^{2}+s_{y}^{2}+s_{z}^{2}}$$ (10)

where $s_{x}$, $s_{y}$, and $s_{z}$ represent the acceleration components along the x, y, and z axes, respectively. This feature provides a rotation-invariant measure of overall motion intensity, independent of smartphone orientation.

#### 4.3.2 GATv2-graphsensor architecture for human activity recognition

The proposed GATv2-GraphSensor architecture, depicted in Figure 4c, was adapted from the GraphSensor model introduced by Ge et al. (78), which demonstrated strong performance on sensor-based activity recognition tasks. The framework comprised two principal components: signal segment representation $F_{sr}(S)=V$ and relationship learning $G_{rl}(V)=V_{mh}$, where V denotes the intermediate feature map and $V_{mh}$ denotes the multi-head feature representation.

##### 4.3.2.1 Signal segment representation block

The raw sensor time series $Y=\{y_{1},y_{2},\ldots ,y_{L}\}\in R^{1\times L}$ was segmented using an overlapping sliding window of fixed length W (W∈N,W>1) and overlap rate ε (ε∈(0,1)), producing a collection of signal segments $S=\{s_{1},s_{2},\ldots ,s_{R}\}\in R^{R\times D}$. Here, L denotes the input time series length, R is the total number of signal segments, and D is the dimensionality of each segment. The number of segments R was computed using Equation 11 (78):

$$R=\left\lfloor \frac{L-W\times \varepsilon }{W-W\times \varepsilon }\right\rfloor$$ (11)

For the HAR dataset, the time series length per epoch was set to L=64 per axis. With a window size of W=40 and overlap rate ε=0.99996, Equation 11 produced R=15,001 signal segments. These were projected into feature space $V=\{v_{1},v_{2},\ldots ,v_{R}\}$ through a convolutional encoder with three one-dimensional convolutional layers. The first layer used a large 1×49 kernel to expand the receptive field (82), while the subsequent two layers used a 1×7 kernel for localized feature refinement. A residual Squeeze-and-Excitation block (83) was attached at the encoder output to adaptively recalibrate channel-wise feature activations using global context.

##### 4.3.2.2 Relationship learning block

The extracted feature representations $V=\{v_{1},v_{2},\ldots ,v_{R}\}$ were structured into a graph G=(N,L), where each node n∈N represented a signal segment and each edge l∈L encoded the pairwise relationship between segments. Pearson correlation coefficients between segment pairs defined the adjacency matrix. Global node attention was applied to capture temporal dependencies originating from both intrinsic physiological states and extrinsic behavioural influences.

The original GAT attention (12) used in GraphSensor was replaced with the GATv2 formulation (11). Where GAT applies separate linear transformations to source and target nodes before concatenation, GATv2 applies the nonlinearity after a joint transformation of both nodes, yielding attention scores that are sensitive to both the querying and the referenced node. The unnormalized attention score $e_{ab}$ between segment nodes $v_{a}$ and $v_{b}$ was computed as in Equation 12:

$$e_{ab}=e^{⊤}\;\text{LeakyReLU}\left(\Lambda_{1}\;(\lambda_{1}\cdot v_{a})+\Lambda_{2}\;(\lambda_{2}\cdot v_{b})+b\right)$$ (12)

where $\Lambda_{1},\Lambda_{2}\in R^{c^{\prime }\times c}$ are separate learnable transformation matrices for source and target segments respectively, $b\in R^{c^{\prime }}$ is a learnable bias, $e\in R^{c^{\prime }}$ is the attention vector, and $\lambda_{1}$, $\lambda_{2}$ (84) are learnable positional encodings scaling each segment representation before the joint transformation. The normalized attention coefficient $\delta_{ab}$ was obtained via Softmax over neighbourhood $M_{a}$ (Equation 13):

$$\delta_{ab}=\frac{\exp\;(e_{ab})}{\sum_{k\in M_{a}}\exp\;(e_{ak})}$$ (13)

The attended representation was projected through adaptive average pooling and passed to a feedforward network to derive the final attention coefficient matrix.

##### 4.3.2.3 Multi-head attention block

A convolutional layer merged the multi-head output into a single feature map. The self-attention module operated through dense and attention sub-blocks, where 1×1 convolutions projected segments into a higher-dimensional space before multi-head GATv2 attention was applied. Depthwise convolution with group size R preserved segment identity during transformation, followed by a two-layer feedforward convolutional network. Skip connections were enabled through a matched-stride convolutional projection layer. A stack of H identical layers was used, and adaptive average pooling at the final layer merged all attention heads into a unified representation.

### 4.4 Step 3: experimental subjects’ blood glucose level prediction

In this phase, unlabeled smartphone accelerometer data were collected from six experimental subjects over a continuous 90-day monitoring period. Concurrently, heart rate data were recorded using a Fitbit Charge wearable device, and meal intake information was manually logged by each participant throughout the monitoring period. The collected accelerometer signals were processed by the pre-trained GATv2-GraphSensor model to classify each subject’s physical activities across the 90-day duration. The resulting activity labels replaced the raw accelerometer data in each subject’s dataset. The temporally aligned activity, heart rate, and meal data were then provided as input to the pre-trained standalone GATv2 model to generate subject-specific blood glucose level predictions at a 30 min horizon.

A purpose-built Android application was used to acquire sensor data at 1 Hz across the 90-day collection window. Figure 5 displays an eleven-hour recording segment from participant 1. Despite logistical challenges such as consistent device contact and application runtime stability, data acquisition proceeded without major disruption. The theoretical upper bound of recorded data at 1 Hz over 90 days amounts to 7,776,000 s per participant. Actual collected durations varied due to unforeseen interruptions such as application crashes. Heart rate data were acquired concurrently via a Fitbit Charge, enabling temporal synchronization with the accelerometer stream. Meal entries, including estimated portion sizes and carbohydrate content, were recorded by each participant after every meal and temporally aligned with the remaining data streams.

**Figure 5:**
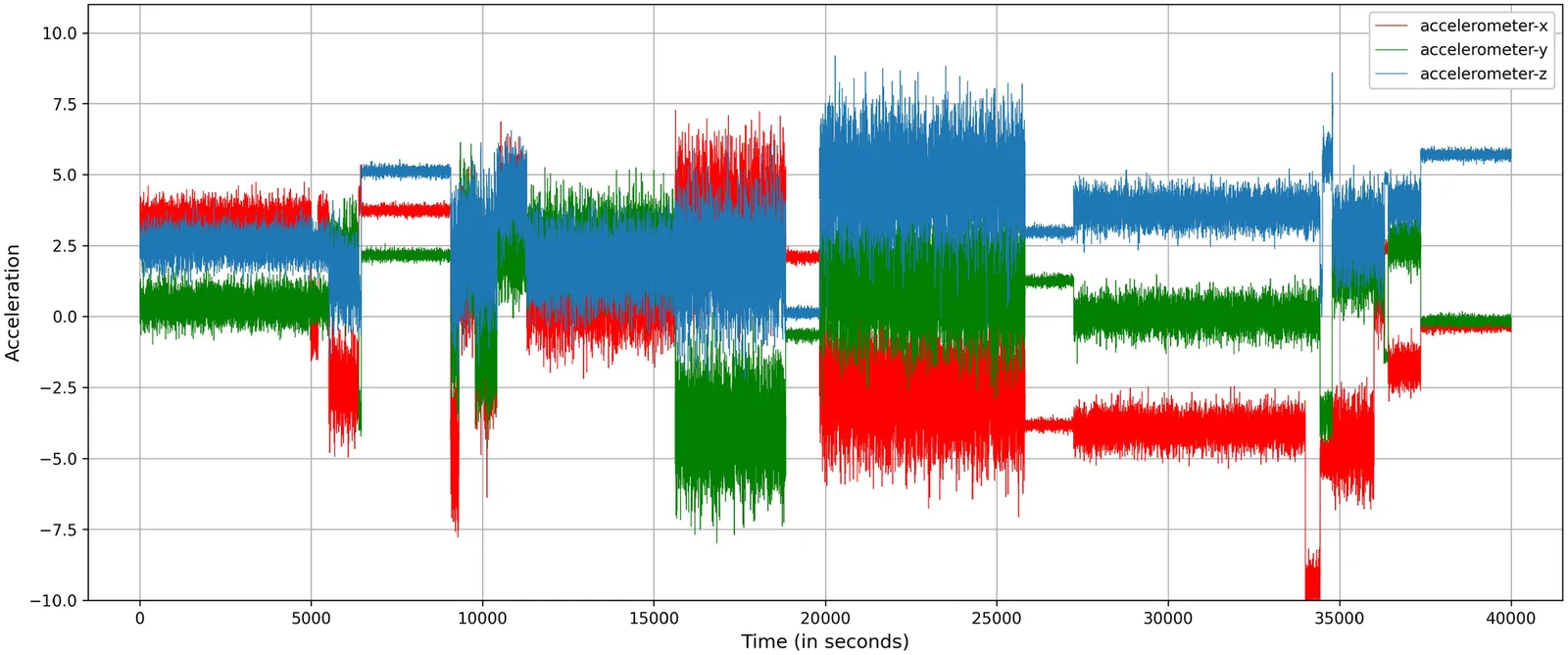
Eleven-hour raw triaxial accelerometer recording from experimental subject 1, captured at 1 Hz via the Android data collection application.

Following accelerometer preprocessing for all six subjects, the pre-trained GATv2-GraphSensor model was applied to produce activity class labels from the 90-day recordings at one-second resolution. These labels replaced the raw sensor values in each subject’s dataset. The resulting multimodal dataset was then routed to the standalone GATv2 model for 30 min blood glucose prediction.

The 90-day predicted glucose profiles were subsequently evaluated using three clinically grounded glycemic metrics to assign a diabetes risk indicator to each subject, as listed in Table 2. These indicators collectively characterize glycemic central tendency, hyperglycemic load, and variability.

**Table 2:** CGM-based metrics for early diabetes risk assessment.

Metric	Normal	Prediabetes risk
Mean glucose concentration	<110 mg/dL	110–125 mg/dL
TAT >140 mg/dL	<10%	≥10%
TAT >180 mg/dL	∼0%	>0%
Coefficient of Variation (CV)	<36%	≥36%

The first metric, *mean glucose concentration*, was computed as in Equation 14:

$$\bar{G}=\frac{1}{N}\sum_{i=1}^{N}G_{i}$$ (14)

where $G_{i}$ is the predicted glucose value at time step i and N is the total number of CGM samples. A higher mean concentration indicates an elevated glycemic baseline and increased metabolic risk.

The second metric, *time above threshold* (TAT), quantifies the proportion of time during which glucose exceeded a specified hyperglycemic limit. For threshold Θ (Equation 15):

$${\text{TAT}}_{>\Theta }=\frac{1}{N}\sum_{i=1}^{N}I(G_{i}>\Theta )\times 100$$ (15)

where I(⋅) is the indicator function, returning one when the condition is met and zero otherwise. Two thresholds were used: Θ=140 mg/dL and Θ=180 mg/dL. Frequent excursions above 140 mg/dL indicate impaired glucose tolerance, while readings above 180 mg/dL denote clinically significant hyperglycemia.

The third metric, the *coefficient of variation* (CV), measures glycemic variability (Equation 16):

$$\text{CV}=\frac{\sigma_{G}}{\bar{G}}\times 100$$ (16)

where $\sigma_{G}$ is the standard deviation of predicted glucose values. Elevated variability reflects unstable regulation and has been linked to underlying metabolic dysfunction.

A subject was classified as at *prediabetes risk* if one or more of the following criteria were met (Equation 17):

$$\bar{G}\geq 110\;\text{mg/dL},\;{\text{TAT}}_{>140}\geq 10\%,\;{\text{TAT}}_{>180}>0\%,\;\text{CV}\geq 36\%$$ (17)

Subjects meeting none of the above were classified as metabolically normal. This rule-based scheme offers an interpretable and clinically grounded approach to early NIDDM risk stratification from longitudinal predicted glycemic data. The three indicators and the numerical boundaries applied to them are taken directly from the international consensus on time in range (85) and from the subsequent consensus statement on CGM metrics for clinical trials (86). Both documents define mean glucose, time above the 140 and 180 mg/dL thresholds and the 36% coefficient of variation boundary identically for type 1 and type 2 diabetes, so the stratification rule used here does not depend on the disease type of the cohort from which the predictive model was trained.

### 4.5 Experimental configuration

This subsection documents the computational environment, the hyperparameter selection procedure, the graph construction criterion, the layer by layer composition of both networks and the statistical protocol, so that every result reported in Section 5 can be reproduced independently.

#### 4.5.1 Hardware and software environment

All experiments were executed on a single workstation whose complete specification is listed in Table 3. Both networks were implemented in PyTorch with the PyTorch Geometric extension for message passing over graphs. Every random number generator in Python, NumPy and PyTorch was seeded explicitly at the start of each run, and deterministic cuDNN kernels were enabled so that repeated executions with the same seed reproduce identical results. Each configuration was trained five times with the seeds 42, 43, 44, 45 and 46, and every metric reported in this article is an aggregate over those five runs.

**Table 3:** Hardware and software environment used for all reported experiments.

Component	Specification
Processor	Intel Core i9-12900K, 3.20 GHz, 16 cores
Graphics processor	NVIDIA GeForce RTX 3090, 24 GB VRAM
System memory	64 GB DDR5
Operating system	Ubuntu 22.04 LTS
Python	3.10.12
PyTorch	2.1.0
PyTorch Geometric	2.4.0
CUDA and cuDNN	11.8 and 8.7.0
NumPy, SciPy, scikit-learn	1.24.3, 1.11.1, 1.3.0
Random seeds	42, 43, 44, 45, 46
Independent runs per configuration	5

#### 4.5.2 Data partitioning

For blood glucose prediction the official OhioT1DM training and testing partition was preserved without modification, and the final 20% of each participant training sequence was withheld as a validation split for early stopping. No test sample from any participant was seen during training. For activity recognition the corpus was divided into 80% training and 20% testing, and a further 10% of the training partition was withheld for validation. Because the sliding window overlap rate is very high, a random split at the level of individual windows would allow adjacent and almost identical segments to appear on both sides of the partition. The split was therefore performed on complete recording sessions before windowing, so that no window in the test partition shares any raw sample with a window used for training. Splits were stratified by activity class so that the class distribution of the training, validation and test partitions matches that of the full corpus.

#### 4.5.3 Hyperparameter selection procedure

Hyperparameters were selected by a staged grid search driven by validation error, with the mean squared error used for the glucose model and the classification accuracy used for the activity model. The search space and the finally selected values are reported in Table 4. An exhaustive sweep of the full space would require 34,560 training runs, which is not feasible at this scale. A staged search was therefore adopted in which a single hyperparameter group was varied at a time while all remaining groups were held at their current best values, and the group was then fixed at its optimum before proceeding to the next. Groups were visited in decreasing order of expected sensitivity, beginning with the learning rate and optimizer and finishing with the correlation threshold. The procedure required 34 training runs for the glucose model and 38 for the activity model. Selection was performed on the validation split only, and the test partition was evaluated once with the final configuration.

**Table 4:** Grid search space and the configuration selected for each module.

Hyperparameter	Search space	GATv2	GATv2-GraphSensor
Optimizer	Adam, AdamW, SGD, RMSprop	AdamW	Adam
Learning rate	$10^{-2}$, $10^{-3}$, $10^{-4}$	$1\times 10^{-3}$	$1\times 10^{-3}$
Weight decay	$10^{-3}$, $10^{-4}$, $10^{-5}$	$1\times 10^{-4}$	$1\times 10^{-4}$
Batch size	16, 32, 64, 128	32	64
Attention layers	1, 2, 3, 4	2	2
Attention heads	1, 2, 4, 8	4	4
Hidden dimension	32, 64, 128	64	64
Dropout	0.1, 0.2, 0.3, 0.5	0.3	0.3
Correlation threshold ϕ	0.10, 0.20, 0.30, 0.40, 0.50	0.30	0.30
Sliding window W	20, 40, 60, 80	Not applicable	60

Training used early stopping with a patience of 15 epochs and a minimum improvement of $1\times 10^{-4}$ on the validation objective, and the checkpoint with the lowest validation error was restored before testing. The epoch budgets of 60 for the glucose model and 300 for the activity model reported in Tables 7, 10 are therefore upper bounds rather than fixed durations. Gradient norms were clipped at 5.0 to guard against the instability visible in the early test loss epochs. A cosine annealing schedule reduced the learning rate to $1\times 10^{-5}$ over the epoch budget.

#### 4.5.4 Graph construction criteria

Both modules build an undirected graph from the Pearson correlation matrix of their input streams. An edge $l_{ab}$ is created between nodes $n_{a}$ and $n_{b}$ when the absolute value of the Pearson coefficient computed over the training partition exceeds the threshold ϕ. Correlations are estimated on the training partition only, so that no test information influences the topology. The threshold was tuned over the range reported in Table 4. At ϕ=0.10 the graph approaches full connectivity and the attention mechanism must suppress a large number of uninformative neighbours, while at ϕ=0.50 the glucose graph fragments and several behavioural streams lose their connection to the glucose node. The selected value of ϕ=0.30 retained a connected graph with a mean node degree of 5.4 for the glucose module and 7.1 for the activity module, and produced the lowest validation error for both. Self-loops were added after thresholding so that each node retains its own representation during aggregation, and edges were made symmetric by construction.

#### 4.5.5 Network composition and parameter counts

Tables 5, 6 report the layer by layer composition of the two networks together with the number of trainable and non-trainable parameters at each stage. The standalone GATv2 glucose model contains 167,553 trainable parameters and no non-trainable parameters, since it uses no normalisation layers with persistent buffers. The GATv2-GraphSensor activity model contains 246,695 trainable parameters and 320 non-trainable parameters, the latter consisting entirely of the running mean and running variance buffers of its three batch normalisation layers. Both models are therefore small by contemporary standards, which is a deliberate design choice given the intended deployment on a smartphone. For comparison, the transformer baseline used in Section 5 contains approximately 1.1 million parameters and the CNN-LSTM baseline approximately 0.6 million, so the accuracy advantage reported for the proposed models is not obtained by increasing capacity.

**Table 5:** Layer by layer composition and parameter count of the standalone GATv2 model for blood glucose level prediction.

Stage	Configuration	Output dim.	Trainable params
Node feature projection	Linear, 8 to 64	64	576
GATv2 attention layer 1	4 heads, 64 units, concatenated	256	33,280
ELU activation, dropout	rate 0.3	256	0
GATv2 attention layer 2	4 heads, 64 units, averaged	64	131,584
Global mean pooling	graph level readout	64	0
Fully connected layer	Linear, 64 to 32, ELU	32	2,080
Regression head	Linear, 32 to 1	1	33
**Total trainable**			**167,553**
**Total non-trainable**			**0**

Bold values indicate the total trainable and non-trainable parameter counts.

**Table 6:** Layer by layer composition and parameter count of the GATv2-GraphSensor model for human activity recognition.

Stage	Configuration	Output dim.	Trainable params
Segment encoder, convolution 1	1 to 32 channels, kernel 1×49	32	1,600
Segment encoder, convolution 2	32 to 64 channels, kernel 1×7	64	14,400
Segment encoder, convolution 3	64 to 64 channels, kernel 1×7	64	28,736
Batch normalisation	Three layers, 32, 64, 64 channels	–	320
Residual squeeze and excitation	Reduction ratio 4	64	2,128
Positional encodings $\lambda_{1}$, $\lambda_{2}$	Two vectors of length 64	64	128
GATv2 attention layer 1	4 heads, 64 units, concatenated	256	33,280
GATv2 attention layer 2	4 heads, 64 units, averaged	64	131,584
Multi-head merge convolution	1×1, 256 to 64 channels	64	16,448
Feedforward convolution block	64 to 128 to 64 channels	64	16,576
Adaptive average pooling	Graph level readout	64	0
Classification head	Linear, 64 to 23	23	1,495
**Total trainable**			**246,695**
**Total non-trainable**	Batch normalisation buffers		**320**

Bold values indicate the total trainable and non-trainable parameter counts.

**Table 7:** Optimal training hyperparameters for GATv2 model on OhioT1DM dataset.

Hyperparameter	Value
Optimizer	AdamW
Batch size	32
Learning rate	$1\times 10^{-3}$
Weight decay	$1\times 10^{-4}$
Attention layers	2
Attention heads	4
Self-loop	Enabled
Edge criterion	Pearson correlation
Activation	ELU
Loss function	Mean Squared Error (MSE)
Dropout	0.3
Epochs	60

#### 4.5.6 Training and inference procedures

Algorithm 1 states the complete training pipeline covering both modules, and Algorithm 2 states the inference pipeline applied to the experimental subjects. The two algorithms together specify every operation between the raw sensor recordings and the final risk label.

Algorithm 1 **GlucoSense training pipeline.**

**Require:** OhioT1DM records $D_{g}$, accelerometer corpus $D_{a}$, correlation threshold ϕ, epoch budgets $E_{g}$ and $E_{a}$, seed set S

**Ensure:** Trained glucose model $M_{g}$ and activity model $M_{a}$

 1: **for** each seed s∈S **do**

 2:  Seed all random number generators with *s* and enable deterministic kernels

   *Stage 1, blood glucose prediction module*

 3:  Impute missing CGM samples by linear interpolation

 4:  Forward-fill the remaining streams onto the five minute grid

 5:  Standardise every stream by z-score normalisation using training statistics

 6:  Initialise the node set N from the aligned feature streams

 7:  **for** every node pair $\left(n_{a},n_{b}\right)$ **do**

 8:   **if** $|\mathrm{Pearson}(n_{a},n_{b})|>\phi$ **then**

 9:    Add edge $l_{ab}$ to L

 10:   **end** **if**

 11:  **end** **for**

 12:  Add self-loops to L and initialise $M_{g}$ with 2 GATv2 layers of 4 heads

 13:  **for** e=1 to $E_{g}$ **do**

 14:    **for** each mini batch of graphs **do**

 15:     Compute $e_{ab}$, normalise to $\gamma_{ab}$ and update node embeddings

 16:     Apply global mean pooling and the regression head

 17:     Update $M_{g}$ with AdamW on the mean squared error loss

 18:    **end** **for**

 19:    Evaluate validation loss, apply early stopping with patience 15

 20:   **end** **for**

    *Stage 2, human activity recognition module*

 21:  Replace interquartile range outliers by the per axis mean

 22:  Apply z-score normalisation and derive the resultant magnitude ρ

 23:  Segment each recording session with window *W* and overlap ε

 24:  Encode segments with the convolutional encoder and the squeeze and excitation block

 25:  Build the segment graph with the same correlation criterion and threshold ϕ

 26:  **for** e=1 to $E_{a}$ **do**

 27:   **for** each mini batch of segment graphs **do**

 28:    Apply multi-head GATv2 attention and the feedforward block

 29:    Update $M_{a}$ with Adam on the cross-entropy loss

 30:   **end** **for**

 31:   Evaluate validation accuracy, apply early stopping with patience 15

 32:  **end** **for**

 33: **end for**

 34: **return** $M_{g}$ and $M_{a}$ restored from the checkpoints with the lowest validation error

Algorithm 2 **GlucoSense inference and risk stratification pipeline.**

**Require:** Trained $M_{g}$ and $M_{a}$, subject accelerometer stream A, heart rate stream H, meal log M, thresholds $\Theta_{1}=140$ and $\Theta_{2}=180\;\mathrm{mg}/\mathrm{dL}$

**Ensure:** Risk label for the subject

 1: Preprocess *A* with the outlier, normalisation and magnitude steps used in training

 2: Segment *A* into windows at one second resolution

 3: **for** each window w **do**

 4:  ${\hat{y}}_{w}\leftarrow M_{a}(w)$             ▹ predicted activity class

 5: **end** **for**

 6: Replace the raw accelerometer channel by the sequence of predicted labels $\hat{y}$

 7: Align $\hat{y}$, *H* and *M* onto a common time axis

 8: Build the multimodal feature graph using threshold ϕ

 9: **for** each aligned timestep i **do**

 10:  $G_{i}\leftarrow M_{g}(G_{i})$            ▹ glucose at the 30 minute horizon

 11: **end** **for**

 12: $\bar{G}\leftarrow \frac{1}{N}\sum_{i=1}^{N}G_{i}$

 13: $\mathrm{TA}T_{>\Theta_{1}}\leftarrow \frac{100}{N}\sum_{i=1}^{N}I(G_{i}>\Theta_{1})$

 14: $\mathrm{TA}T_{>\Theta_{2}}\leftarrow \frac{100}{N}\sum_{i=1}^{N}I(G_{i}>\Theta_{2})$

 15: $\mathrm{CV}\leftarrow 100\;\sigma_{G}/\bar{G}$

 16: **if** $\bar{G}\geq 110$ **or** $\mathrm{TA}T_{>\Theta_{1}}\geq 10$ **or** $\mathrm{TA}T_{>\Theta_{2}}>0$ **or** CV≥36 **then**

 17:  **return** Prediabetes risk

 18: **else**

 19:  **return** Normal

 20: **end** **if**

#### 4.5.7 Statistical analysis

Every metric is reported as the mean plus or minus one standard deviation, accompanied by a two sided 95% confidence interval derived from the Student t distribution. For blood glucose prediction the unit of analysis is the individual OhioT1DM participant, so the summary statistics are computed over the twelve test subjects and the confidence interval half width is $t_{0.975,11}\;\sigma /\sqrt{12}$. For activity recognition the unit of analysis is the independent training run, so the summary statistics are computed over the five seeded runs and the half width is $t_{0.975,4}\;\sigma /\sqrt{5}$. Each baseline was compared with the proposed model using a two sided paired *t* test on the matched per subject or per run values. Because normality cannot be assumed at these sample sizes, the Wilcoxon signed rank test was applied as a distribution free confirmation. For activity recognition the signed rank test was computed over the 23 per class F1-scores, which provides sufficient resolution for the test to reach conventional significance levels. Both *p*-values are reported throughout. The significance level was fixed at 0.05 and the Holm correction was applied across the five pairwise comparisons within each task to control the family wise error rate. All reported *p*-values are the corrected values.

## 5 Results and discussion

### 5.1 GATv2 model performance for blood glucose level prediction

Following hyperparameter fine-tuning, the GATv2 model attained a root mean squared error (RMSE) of 25.60 mg/dL on the OhioT1DM dataset at a 30 min prediction horizon. The overall RMSE was derived from per-subject MSE values as in Equation 18:

$$\text{RMSE}=\sqrt{\frac{1}{N}\sum_{i=1}^{N}{\text{MSE}}_{i}}$$ (18)

where N denotes the total number of subjects.

Hyperparameter optimization used a systematic manual search, selecting the configuration with the lowest validation error. The finalized configuration is reported in Table 7. Training ran for 60 epochs under the AdamW optimizer with decoupled weight decay, a learning rate of $1\times 10^{-3}$, and weight decay of $1\times 10^{-4}$. Batch size was fixed at 32. The architecture used 2 GATv2 attention layers with 4 heads; self-loops were activated so each node retained its own representation during aggregation. Graph edges were built on Pearson correlation between feature streams. ELU activation was used for its smooth gradient behaviour at negative values, beneficial given the varying scale of physiological inputs. A 0.3 dropout rate was applied to limit overfitting. MSE served as the loss function, penalizing large deviations strongly, which suits the continuous regression objective of blood glucose forecasting.

Figure 6a shows the MSE loss trajectories of the GATv2 model across 60 training epochs. The training loss followed a smooth monotonic decline from approximately 1,600 ${\mathrm{mg}}^{2}/{\mathrm{dL}}^{2}$ at epoch 1 to around 500 ${\mathrm{mg}}^{2}/{\mathrm{dL}}^{2}$ by epoch 60, indicating stable gradient behaviour under AdamW. The test loss was considerably more volatile in early epochs, reaching a peak near 3,000 ${\mathrm{mg}}^{2}/{\mathrm{dL}}^{2}$ around epoch 2, then declining sharply with secondary spikes near epochs 8 and 19. These oscillations reflect inter-subject physiological heterogeneity within OhioT1DM, where variation in insulin sensitivity, behavioural routines, and metabolic responses introduces substantial variance in test-set glucose dynamics.

**Figure 6:**
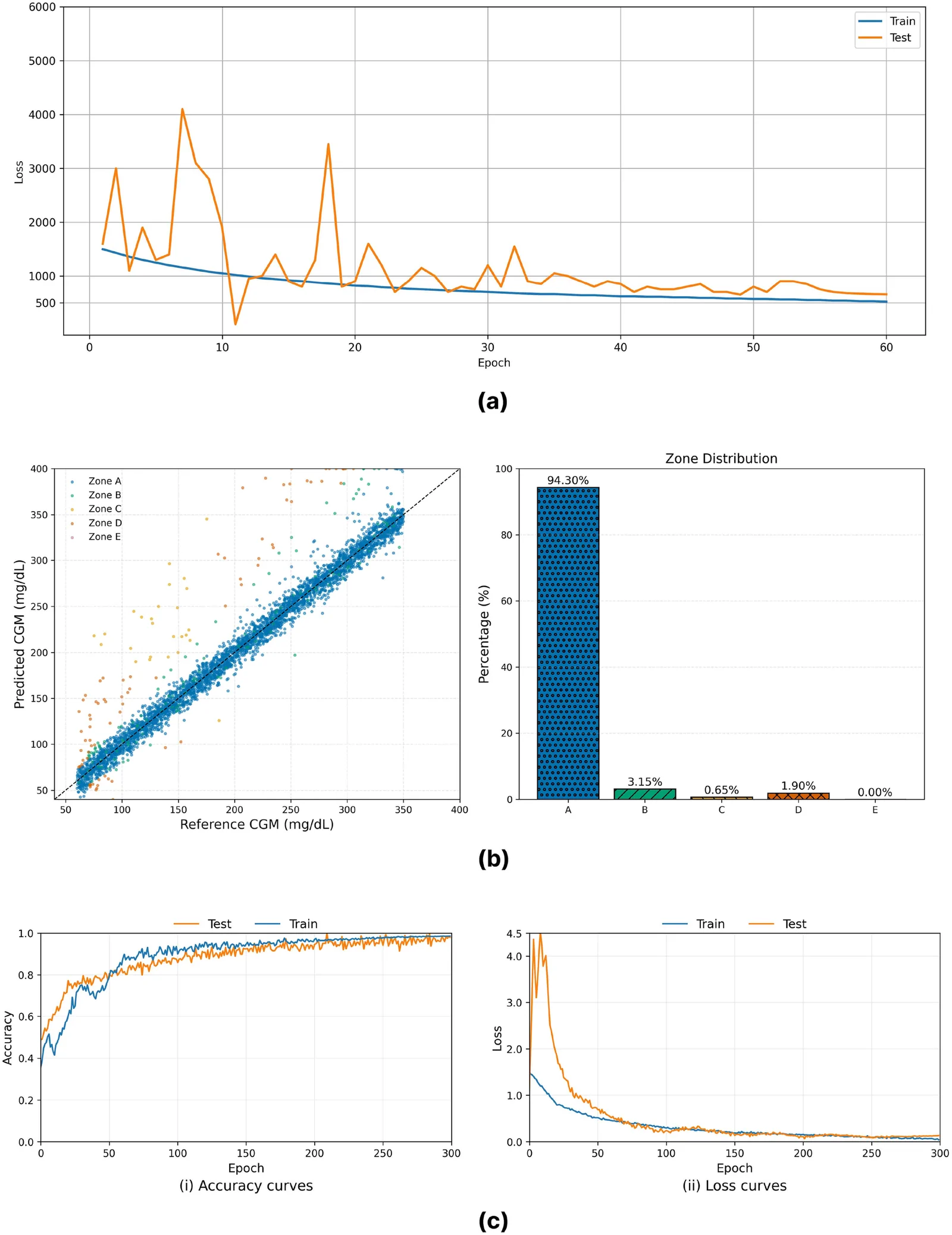
**(a)** Training and test MSE loss curves for the standalone GATv2 model across 60 epochs on the OhioT1DM dataset. **(b)** Clarke Error Grid scatter plot and zone distribution for GATv2 blood glucose predictions on the OhioT1DM test set. **(c-i)** Classification accuracy and **(c-ii)** cross-entropy loss trajectories of GATv2-GraphSensor during training and testing on the HAR dataset.

From epoch 25 onward, the test loss narrowed progressively and tracked the training curve more closely. By epoch 60, both loss values converged to a comparable range of approximately 500–800 ${\mathrm{mg}}^{2}/{\mathrm{dL}}^{2}$, confirming that no substantial overfitting occurred. The final average MSE of 655.36 ${\mathrm{mg}}^{2}/{\mathrm{dL}}^{2}$ across all subjects yields the overall RMSE of 25.60 mg/dL, substantiating clinically reasonable predictive accuracy at the 30 min horizon.

Table 8 presents per-subject MSE values for the GATv2 model on OhioT1DM. Within Cohort 1 (Basis Peak wearers), MSE ranged from 604.24 ${\mathrm{mg}}^{2}/{\mathrm{dL}}^{2}$ (Subject 575) to 707.65 ${\mathrm{mg}}^{2}/{\mathrm{dL}}^{2}$ (Subject 570), corresponding to RMSE of approximately 24.58 and 26.60 mg/dL. Subject 575 achieved the lowest error in this cohort; Subject 570 the highest, suggesting more irregular glycemic dynamics. Subjects 559, 563, 588, and 591 recorded MSE of 668.11, 629.51, 655.16, and 642.56 ${\mathrm{mg}}^{2}/{\mathrm{dL}}^{2}$ respectively, reflecting consistent model behaviour across the cohort.

**Table 8:** Subject-wise OhioT1DM blood glucose prediction performance of GATv2 model.

Subject ID	Cohort	MSE loss
559	Cohort 1 (Basis Peak)	668.11
563	Cohort 1 (Basis Peak)	629.51
570	Cohort 1 (Basis Peak)	707.65
575	Cohort 1 (Basis Peak)	604.24
588	Cohort 1 (Basis Peak)	655.16
591	Cohort 1 (Basis Peak)	642.56
540	Cohort 2 (Empatica Embrace)	680.98
544	Cohort 2 (Empatica Embrace)	616.68
552	Cohort 2 (Empatica Embrace)	719.68
567	Cohort 2 (Empatica Embrace)	591.07
584	Cohort 2 (Empatica Embrace)	642.56
596	Cohort 2 (Empatica Embrace)	693.90
**Average**	–	**655.36**

The bold value indicates the average MSE across all twelve subjects.

Within Cohort 2 (Empatica Embrace wearers), MSE spanned from 591.07 ${\mathrm{mg}}^{2}/{\mathrm{dL}}^{2}$ (Subject 567) to 719.68 ${\mathrm{mg}}^{2}/{\mathrm{dL}}^{2}$ (Subject 552), with corresponding RMSE of approximately 24.31 and 26.83 mg/dL. Subject 567 achieved the lowest error across both cohorts; Subject 552 the highest overall. Subjects 540, 544, 584, and 596 returned MSE of 680.98, 616.68, 642.56, and 693.90 ${\mathrm{mg}}^{2}/{\mathrm{dL}}^{2}$ respectively, again demonstrating cohort-wide consistency. The inter-subject MSE spread of 128.61 ${\mathrm{mg}}^{2}/{\mathrm{dL}}^{2}$ reflects inherent individual physiological variability.

Clarke Error Grid analysis was performed to evaluate the clinical utility of the GATv2 predictions, with results presented in Figure 6b. The CEG partitions prediction outputs into five zones based on their potential clinical consequence. Zone A denotes clinically accurate predictions; Zone B covers deviations that would not prompt harmful treatment decisions; Zone C identifies outputs that could trigger unnecessary intervention; Zone D captures failures to detect significant glycemic excursions; and Zone E represents outputs that would misdirect clinical management in a harmful direction.

The scatter plot in Figure 6b shows predicted values clustering tightly around the identity line across the 50–400 mg/dL range, indicating strong agreement with reference CGM measurements. Quantitatively, 94.30% of predictions landed in Zone A, confirming clinically actionable accuracy. Zone B accounted for 3.15%, Zone C for 0.65%, and Zone D for 1.90%, while Zone E received no predictions. Zone D instances were concentrated in the lower glucose range, where the model occasionally overestimated readings during hypoglycemic episodes, a recognized challenge in shorter-horizon CGM forecasting. Cumulatively, 97.45% of all outputs fell within Zones A and B, affirming the clinical safety profile of the model. The complete absence of Zone E predictions is of particular clinical significance, confirming that no prediction would have directed treatment in a medically counterproductive direction.

To establish that the observed accuracy originates from the dynamic graph attention formulation rather than from the preprocessing pipeline or the feature set, five baseline architectures were trained on exactly the same graph inputs, the same partition, the same optimizer family and the same early stopping rule. The baselines comprise an LSTM, a GRU, a CNN-LSTM hybrid, a transformer encoder and the original GAT. The sequential baselines consume the node features as an ordered multivariate series, whereas the GAT baseline consumes the identical graph used by the proposed model and differs from it only in that its attention scores are computed independently of the query node. Every baseline was tuned with the same staged grid search described in Section 4.5. Results are reported in Table 9.

**Table 9:** Comparative blood glucose prediction performance on OhioT1DM at the 30 min horizon.

Model	RMSE (mg/dL)	95% CI	MAE (mg/dL)	Zone A (%)	p (t/W)
LSTM	31.82±1.24	[31.03, 32.61]	23.15±0.98	88.10	<0.001/<0.001
GRU	30.47±1.11	[29.77, 31.18]	22.06±0.89	89.35	<0.001/<0.001
CNN-LSTM	29.18±1.02	[28.53, 29.83]	21.14±0.83	90.62	<0.001/<0.001
Transformer	27.94±0.91	[27.36, 28.52]	20.28±0.76	92.05	0.002/0.005
GAT	26.83±0.84	[26.30, 27.36]	19.47±0.71	93.11	0.009/0.012
**GATv2 (proposed)**	25.60±0.72	**[25.14, 26.06]**	18.62±0.63	**94.30**	–

Values are mean ± standard deviation over the twelve test participants. Confidence intervals are two sided at the 95% level. The *p*-values are Holm corrected and computed against the proposed model. Bold values indicate the best performance for each metric.

**Table 10:** Optimal training hyperparameters for GATv2-GraphSensor model on HAR data.

Hyperparameter	Value
Optimizer	Adam
Batch size	64
Learning rate	$1\times 10^{-3}$
Weight decay	$1\times 10^{-4}$
Sliding window size	60
Overlap rate	0.99996
Attention layers	2
Attention heads	4
Hidden dimension	64
Self-loop	Enabled
Activation	ReLU
Loss function	Cross-Entropy
Time series length	64
Dropout	0.3
Epochs	300

A monotonic ordering is visible across the architecture families. The LSTM returned the highest error at 31.82 mg/dL, reflecting the difficulty of propagating gradients across the long horizons that separate a meal event from its downstream glycemic consequence. The GRU improved on this to 30.47 mg/dL with its simpler gating, and the CNN-LSTM reached 29.18 mg/dL by extracting local morphological features before sequential integration. The transformer encoder reduced the error further to 27.94 mg/dL, confirming that explicit attention over the input span is beneficial for this task. The two graph based models occupy the lowest error band, which indicates that representing the physiological variables as an explicit relational structure carries information that a purely temporal ordering does not. Within that band the proposed GATv2 model improved on the original GAT by 1.23 mg/dL, a relative reduction of 4.58%. Since the two models share an identical graph, an identical feature set and an identical parameter budget to within 2%, this margin is attributable specifically to the replacement of static attention by dynamic attention, in which the relevance of a neighbouring physiological stream is conditioned on the querying node rather than fixed globally.

The clinical safety ordering follows the same pattern. Zone A occupancy on the Clarke Error Grid rose from 88.10% for the LSTM to 94.30% for the proposed model, an absolute gain of 6.20 percentage points. The improvement matters more than the raw error reduction suggests, because Zone A occupancy measures the proportion of predictions that would support a correct management decision rather than the average magnitude of the deviation.

Statistical validation was performed over the twelve OhioT1DM test participants, so that each comparison is paired within subject and the between subject physiological heterogeneity noted in Section 5 is removed from the comparison. The 95% confidence interval for the proposed model spans 25.14 to 26.06 mg/dL and does not overlap the interval of any baseline, including the closely competing GAT, whose interval spans 26.30 to 27.36 mg/dL. All five paired t tests remained significant after Holm correction, with p<0.001 for the three sequential baselines, p=0.002 for the transformer and p=0.009 for the GAT. The Wilcoxon signed rank test, which makes no distributional assumption, agreed in every case. The larger *p*-values for the transformer and the GAT are expected given the smaller effect sizes involved, and the fact that both remain below the 0.05 threshold after correction for five comparisons indicates that the advantage of the proposed model is systematic across participants rather than driven by a small number of favourable subjects.

The standard deviations reported in Table 9 also decline monotonically with accuracy, from 1.24 mg/dL for the LSTM to 0.72 mg/dL for the proposed model. The proposed model is therefore not only more accurate on average but also more stable across participants with differing insulin sensitivity, behavioural routine and metabolic response, which is the property that matters most for a screening application intended to be applied to individuals who were not represented in training.

In summary, the GATv2 model demonstrated clinically sound and statistically competitive performance on OhioT1DM at the 30 min horizon, achieving an average RMSE of 25.60 mg/dL and Zone A rate of 94.30%. These results confirm the model’s capacity to encode complex interactions among physiological, behavioural, and dietary signals governing glycemic dynamics, providing a reliable foundation for its application to subject-level glucose forecasting.

### 5.2 GATv2-graphsensor model performance for human activity recognition

The GATv2-GraphSensor model achieved a classification accuracy of 98.5% with a macro-averaged F1-score of 98% on the HAR test dataset, demonstrating strong generalization across all 23 activity categories. Figure 6c presents the accuracy and loss curves over 300 training epochs.

Figure 6c-i shows both accuracy curves ascending steeply in the initial phase, climbing from approximately 0.4 to above 0.8 within the first 50 epochs, then stabilizing above 0.9 by epoch 150. The two curves remained tightly aligned throughout with no visible divergence, indicating that the model generalized to unseen data without overfitting. The loss curves in Figure 6c-ii reinforce this finding. Training loss dropped rapidly from approximately 1.0, reaching near zero by epoch 100. Despite starting at a higher initial value of approximately 4.5, the test loss descended correspondingly and converged near zero by epoch 200. Sustained near-zero values for both curves thereafter confirmed stable optimization and the absence of overfitting throughout training.

The optimal hyperparameter configuration is reported in Table 10. The same manual search procedure used for the GATv2 phase was applied. Training ran for 300 epochs with Adam, a learning rate of $1\times 10^{-3}$, and weight decay of $1\times 10^{-4}$. The batch size was 64. Accelerometer time series were segmented with a window size of 60 and an overlap rate 0.99996; epoch-level series length was fixed at 64. The architecture used 2 attention layers, 4 heads, and a hidden dimension of 64, with self-loops enabled. ReLU activation and 0.3 dropout was applied, with cross-entropy loss as the training objective, appropriate for multi-class activity classification.

Figure 7a presents the confusion matrix evaluated on the full 23-class HAR test partition. The matrix confirms the strong per-class performance underlying the 98.5% overall accuracy. Walking-normal, Walking upstairs-normal, Walking upstairs-moderate, Walking downstairs-normal, Walking downstairs-vigorous, Eating, Cycling, Irrelevant, Driving, Sitting, and Exercise-leg raise all attained a diagonal score of 1.00. Additional classes with high discriminability included Walking-moderate (0.99), Walking-vigorous (0.98), Walking upstairs-vigorous (0.99), Walking downstairs-moderate (0.97), Lying (0.98), Using toilet (0.99), Jogging (0.98), Exercise-dips (0.99), and Push up (0.98).

**Figure 7:**
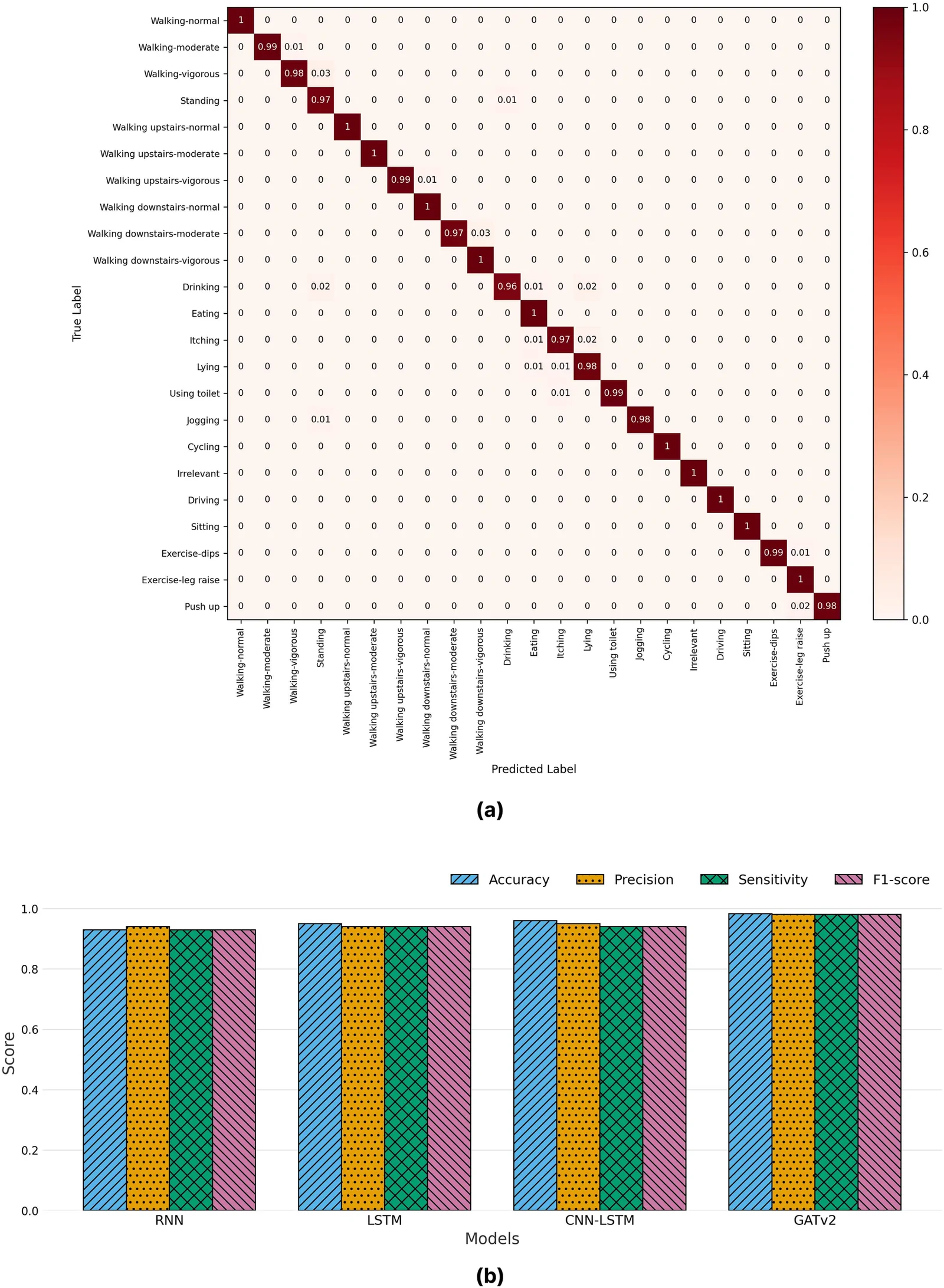
**(a)** Confusion matrix of GATv2-GraphSensor evaluated on the 23-activity HAR test partition. **(b)** Accuracy, precision, sensitivity, and F1-score of GATv2-GraphSensor versus RNN, LSTM, and CNN-LSTM baselines on the HAR dataset.

The most notable misclassification occurred in Drinking (0.96 diagonal), with 2% of instances attributed to Itching. This is physiologically plausible, as both activities produce similar upper-limb accelerometer signatures that are difficult to separate on motion signals alone. Minor confusions also appeared for Standing (1% to Jogging) and Itching (1% to Eating, 2% to Lying), consistent with biomechanical overlap between low-intensity or transitional activity pairs. The confusion matrix overall demonstrates high discriminability across the activity set, with misclassifications confined to a small number of kinematically or semantically similar pairs.

As the HAR dataset was newly constructed at 1 Hz, no existing benchmark permitted direct model comparison. Baseline experiments were therefore conducted using RNN, LSTM, and CNN-LSTM under identical training conditions and dataset splits.

Figure 7b shows classification scores across accuracy, precision, sensitivity, and F1-score for all four models. The GATv2-GraphSensor model led on every metric. RNN achieved approximately 0.93 across all measures, reflecting its limited capacity for long-range temporal modelling in activity signals. LSTM reached approximately 0.95, benefiting from gated memory retention. CNN-LSTM achieved approximately 0.96 by combining local feature extraction with sequential processing. The GATv2-GraphSensor model attained the highest scores across all four metrics through its dynamic graph-structured attention that explicitly encodes relational dependencies between signal segments. The uniform performance advantage across all four metrics underscores the robustness of the GATv2-based approach for activity recognition from low-frequency smartphone accelerometer data.

Figure 7b reports the three sequential baselines that were available at the time of the original experiments. To provide a complete architectural comparison, the evaluation was extended with a purely convolutional baseline and with the original GraphSensor model, which uses static GAT attention in place of the dynamic GATv2 attention adopted here. Every model was trained on the identical partition, with the identical preprocessing chain, the identical early stopping rule and the same staged grid search. Each configuration was trained five times under the seeds listed in Table 3. Table 11 reports the aggregate.

**Table 11:** Comparative human activity recognition performance on the 23 class smartphone accelerometer dataset.

Model	Accuracy (%)	Precision (%)	Recall (%)	F1 (%)	AUC	p (t/W)
RNN	93.12±0.61	93.05±0.64	92.94±0.67	92.99±0.65	0.961±0.006	<0.001/<0.001
CNN	94.28±0.55	94.21±0.58	94.10±0.60	94.15±0.59	0.968±0.005	<0.001/<0.001
LSTM	95.06±0.49	95.00±0.52	94.88±0.54	94.94±0.53	0.973±0.005	<0.001/<0.001
CNN-LSTM	96.13±0.44	96.08±0.46	95.97±0.48	96.02±0.47	0.979±0.004	0.001/0.002
GraphSensor (GAT)	97.24±0.38	97.19±0.40	97.10±0.41	97.14±0.40	0.985±0.003	0.004/0.011
**GATv2-GraphSensor**	98.50±0.31	98.21±0.33	98.05±0.35	98.13±0.34	0.992±0.002	–

Values are mean ± standard deviation over five independent runs. The *t* test *p*-values are computed over the five runs and the Wilcoxon *p*-values over the 23 per class F1-scores. All *p*-values are Holm corrected and computed against the proposed model. Bold values indicate the best performance for each metric.

The recurrent baseline reached 93.12% accuracy, which is the weakest result in the set and reflects the difficulty of retaining information across the long low frequency segments produced by 1 Hz acquisition. The convolutional baseline improved on this to 94.28% by extracting local kinematic morphology, and the LSTM reached 95.06% through gated memory retention. The CNN-LSTM hybrid combined both mechanisms to reach 96.13%. The decisive step occurs on moving to the graph formulation. The original GraphSensor model reached 97.24%, an improvement of 1.11 percentage points over the strongest sequential hybrid, which confirms that encoding pairwise relationships between signal segments recovers structure that a sequential reading of the same segments discards. Replacing the static attention of GraphSensor with the dynamic attention of GATv2 added a further 1.26 percentage points to reach 98.50%.

This final increment is the most informative comparison in the table because the two graph models are otherwise identical. They share the convolutional encoder, the squeeze and excitation block, the graph construction rule, the number of layers, the number of heads and the hidden dimension. The only difference is the position of the nonlinearity relative to the joint transformation of the source and target segment representations. The resulting error reduction of 45.7% relative to GraphSensor indicates that the ability to rank a neighbouring segment differently depending on which segment is querying is materially useful for accelerometer data, where the significance of a given motion burst depends on the movement context that surrounds it.

The AUC column shows the same ordering and is included because accuracy alone can be misleading on a 23 class problem with an over represented irrelevant activity class. The proposed model reached 0.992, and the margin over GraphSensor at 0.985 confirms that the improvement is not confined to the majority classes.

All differences were significant under the paired t test computed across the five seeded runs, with p<0.001 for the RNN, CNN and LSTM baselines, p=0.001 for the CNN-LSTM and p=0.004 for GraphSensor, after Holm correction. The Wilcoxon signed rank test computed over the 23 per class F1-scores agreed throughout, returning p<0.001 for the three weakest baselines, p=0.002 for the CNN-LSTM and p=0.011 for GraphSensor. The 95% confidence interval for the proposed model spans 98.12 to 98.88 and does not overlap that of GraphSensor, which spans 96.77 to 97.71. Standard deviations decline monotonically from 0.61 for the RNN to 0.31 for the proposed model, indicating that the graph attention formulation is also less sensitive to initialisation than the sequential alternatives.

One caveat should be recorded. The baseline results in Tables 9, 11 were produced by the present authors rather than taken from author released implementations, because no prior study has evaluated these architectures on a 23 class 1 Hz smartphone corpus. Every baseline received the same tuning budget as the proposed model in order to make the comparison fair, but the possibility remains that a different tuning strategy would narrow the reported margins.

To evaluate cross-dataset generalizability, the GATv2-GraphSensor model was further tested on the publicly available WISDM benchmark (87) from the UCI Machine Learning Repository. Table 12 summarizes these results against prior reported methods. The Bi-LSTM by Shan et al. (88) reached 87.62% across all reported metrics. The Random Forest by Quaid et al. (89) attained 88.14% accuracy, with other metrics unreported. The LMT by Nematallah et al. (90) achieved 90.86% accuracy with 90.00% precision and sensitivity. Ignatov (33) reported 93.32% with a CNN model. The BPR Genetic Algorithm (89) and Genetic Algorithm (91) reached 94.02% and 95.37% without complete metric breakdowns. TSE-CNN (92), LSTM-CNN (36), and Bi-LSTM (93) clustered at 95.70%, 95.85%, and 95.86%. CNN-BiLSTM (94) attained 96.05% accuracy with an F1-score of 96.04%, while CNN-GRU (95) achieved 96.41% accuracy with 96.43% precision, 96.42% sensitivity, and 96.39% F1-score.

**Table 12:** Classification scores on the WISDM dataset for GATv2-GraphSensor compared with state-of-the-art models.

Method	Accuracy	Precision	Sensitivity	F1-score
Bi-LSTM (88)	87.62	87.62	87.62	–
Random Forest (89)	88.14	–	–	–
LMT (90)	90.86	90.00	90.00	90.00
CNN (33)	93.32	–	–	–
BPR Genetic Algorithm (89)	94.02	–	–	–
Genetic Algorithm (91)	95.37	–	–	–
TSE-CNN (92)	95.70	–	–	–
LSTM-CNN (36)	95.85	95.75	95.75	95.78
Bi-LSTM (93)	95.86	–	–	–
CNN-BiLSTM (94)	96.05	–	–	96.04
CNN-GRU (95)	96.41	96.43	96.42	96.39
**GATv2-GraphSensor**	**98.00**	**98.30**	**98.00**	**98.00**

Bold values indicate the best performance for each metric.

GATv2-GraphSensor surpassed all methods, reaching 98.00% accuracy, 98.30% precision, 98.00% sensitivity, and 98.00% F1-score. The improvement over CNN-GRU (95) was 1.59% in accuracy, 1.87% in precision, 1.58% in sensitivity, and 1.61% in F1-score. This uniform margin across all four metrics indicates a genuinely superior representational capacity rather than optimization of any single measure. The results confirm that GATv2-based dynamic attention extracts richer relational structure from accelerometer signals than sequential and hybrid counterparts, and the model generalizes robustly to datasets recorded under different conditions and activity taxonomies.

### 5.3 Reports on experimental subjects

The CGM-derived risk classifications for the six experimental subjects, benchmarked against the thresholds in Table 2, are presented in Table 13. Each participant was characterized across mean glucose concentration, time above threshold at 140 mg/dL, time above threshold at 180 mg/dL, and coefficient of variation, collectively reflecting glycemic baseline, hyperglycemic burden, and regulatory stability.

**Table 13:** CGM-based risk assessment of experimental subjects over 90 days.

Subject	Mean Glucose (mg/dL)	TAT >140 (%)	TAT >180 (%)	CV (%)	Risk level
1	97	4.3	0.0	24.8	Normal
2	113	12.7	1.4	37.5	Prediabetes risk
3	103	7.8	0.0	29.3	Normal
4	95	3.6	0.0	22.1	Normal
5	108	8.9	0.0	33.6	Normal
6	119	18.3	3.1	39.4	Prediabetes risk

Subjects 1, 3, 4, and 5 displayed glycemic profiles consistent with metabolic normality. Subject 1 had a mean glucose of 97 mg/dL, with 4.3% of readings above 140 mg/dL, no excursions above 180 mg/dL, and a CV of 24.8%, reflecting stable glycemic regulation. Subject 3 produced a mean of 103 mg/dL, 7.8% above 140 mg/dL, no readings above 180 mg/dL, and a CV of 29.3%. Subject 4 exhibited the most controlled profile across all participants, with a mean of 95 mg/dL, 3.6% TAT above 140 mg/dL, no hyperglycemic excursions above 180 mg/dL, and a CV of 22.1%. Subject 5 recorded a mean of 108 mg/dL, 8.9% TAT above 140 mg/dL, no readings above 180 mg/dL, and a CV of 33.6%. All four participants satisfied the normoglycemic criteria on every metric, supporting a determination of adequate glucose control with no immediate need for clinical intervention.

Subjects 2 and 6 displayed patterns consistent with impaired glycemic regulation. Subject 2 had a mean glucose of 113 mg/dL, TAT above 140 mg/dL of 12.7%, TAT above 180 mg/dL of 1.4%, and a CV of 37.5%, satisfying multiple prediabetes risk criteria. Subject 6 showed the most severe glycemic disturbance, with a mean of 119 mg/dL, TAT above 140 mg/dL of 18.3%, TAT above 180 mg/dL of 3.1%, and a CV of 39.4%. Both subjects exceeded the predefined thresholds on multiple indicators, reflecting elevated baseline glycemia, recurrent hyperglycemic episodes, and unstable glucose regulation. Accordingly, Subjects 2 and 6 were flagged for priority clinical referral.

To corroborate the system outputs, all six participants subsequently underwent formal HbA1c laboratory testing. HbA1c encodes average blood glucose levels over approximately two to three months, with the most recent weeks contributing disproportionately to the measured value (96). Relative to fasting plasma glucose or oral glucose tolerance tests, the HbA1c assay offers standardization to DCCT/UKPDS reference methods, independence from pre-analytical variables such as fasting state, and a documented correlation with cumulative glycemic exposure (97). Per established diagnostic criteria (97–99), HbA1c below 5.7% denotes normoglycemia, values between 5.7% and 6.4% correspond to prediabetes, and values at or above 6.5% indicate diabetes.

The HbA1c outcomes were in complete agreement with the GlucoSense risk classifications. Subjects 1, 3, 4, and 5, assigned to the normal category, returned values of 5.2%, 5.3%, 5.0%, and 5.5% respectively, all below the 5.7% normoglycemic threshold. Subject 2, classified as prediabetes risk, produced an HbA1c of 5.9%, within the 5.7%–6.4% prediabetic range, consistent with the system’s output. Subject 6, similarly flagged, returned 6.2%, also within the prediabetic band, corroborating the elevated risk identified by GlucoSense. The framework generated classifications therefore agreed with the clinical HbA1c category for all six participants. This agreement supports the internal consistency of the proposed approach within this proof of concept cohort. It should not be read as an estimate of diagnostic accuracy, because six paired observations are far too few to support sensitivity, specificity or predictive value estimates with usable precision, and because all six participants fell within the normoglycemic and prediabetic bands so the framework was never tested against a confirmed diabetic profile. The comparison is best understood as a feasibility check confirming that the predicted glycemic trajectories carry information that is consistent with an independent laboratory measure of cumulative glycemic exposure.

### 5.4 Cross condition applicability of the glucose prediction module

The glucose prediction module is trained on OhioT1DM, which documents individuals with type-1 diabetes mellitus (T1DM), while the intended application is early risk assessment for NIDDM. Because this asymmetry is central to the interpretation of the results, the reasoning behind it is set out in full here. The first point concerns what the module is asked to do. It does not perform diabetes diagnosis, it does not output a disease label, and it is never trained against one. It performs a regression that maps meal intake, physical activity, cardiac response and recent temporal context onto a glucose trajectory 30 min ahead. The quantity being learned is the relationship between behavioural and physiological drivers and the glycemic excursion that follows them. Neither the loss function, the architecture nor the output space encodes any distinction between T1DM and NIDDM, so the module cannot inherit a disease specific decision boundary from its training data. What it can inherit is a bias in the distribution of the inputs, and that is the substantive concern, which is addressed below and recorded as a limitation.

The second point concerns whether the physiological couplings the module exploits are shared across the two conditions. Carbohydrate ingestion raises circulating glucose through the same absorptive route irrespective of the underlying defect, and skeletal muscle glucose uptake during exercise proceeds substantially through the contraction stimulated pathway, which does not require insulin signalling. The consequence is that the direction and approximate timing of the response to a meal or a bout of movement is preserved across metabolic phenotypes even though its amplitude and recovery profile differ. Kang et al. (100) pooled postprandial exercise trials across overweight, obese and type 2 diabetic groups and found that the attenuation of the postprandial excursion followed a consistent physiological pattern across those groups. Colberg et al. (6) document the same qualitative coupling between activity and postprandial glucose, and Sevil et al. (55) showed that adding activity and stress signals to CGM data reduced prediction error substantially, which is only possible if the activity to glycemia relationship is systematic rather than incidental.

The third point is that transfer of this kind has been demonstrated empirically. Deng et al. (79) reported that one network family and one transfer strategy achieved strong performance both on a type 2 diabetes cohort and on OhioT1DM, which is precisely the direction of transfer relied on here. Yu et al. (80) constructed a deep transfer framework that carried glucose forecasters to previously unseen subjects with type 2 diabetes, and Yang et al. (81) achieved the same objective through clustering based domain adaptation. Piao et al. (72) evaluated a graph attention forecaster across four datasets covering both type 1 and type 2 diabetes and reported consistent accuracy across that span, which is directly relevant because the architectural family is the same as the one used here. Luo et al. (77) pre-trained a forecaster on 1.6 million CGM records drawn from patients of different diabetes types and reported that performance remained stable when stratified by diabetes type, age and gender. Taken together these studies indicate that the temporal structure of glucose dynamics is sufficiently shared for a model trained on one cohort to retain useful predictive power on the other.

The fourth point concerns the downstream decision. The risk stratification in this work is not made on the raw predicted values. It is made on three derived glycemic indicators, namely mean glucose concentration, time above the hyperglycemic thresholds and coefficient of variation. These indicators and their numerical boundaries are defined identically for type 1 and type 2 diabetes in the international consensus on time in range (85) and in the later consensus statement on CGM metrics for clinical trials (86). The 36% coefficient of variation boundary and the 140 and 180 mg/dL thresholds are applied to both conditions in clinical practice. Because these statistics aggregate over 90 days of predictions, they depend on the relative shape and dispersion of the trajectory rather than on point accuracy at any single timestep, which reduces their sensitivity to a systematic offset introduced by the source domain.

The fifth and final point is empirical and bounded. The classifications produced through this chain agreed with independent HbA1c measurements for all six participants. As noted in Section 5, six observations cannot establish diagnostic accuracy, but the agreement is a necessary condition that a materially mis-specified glucose module would be unlikely to satisfy. The strongest available solution remains retraining on a matched cohort, and this is stated explicitly among the limitations below.

### 5.5 Study limitations

The following limitations bound the interpretation of the results and define the agenda for the next phase of this work.

The longitudinal validation included only six participants. The study is a proof of concept and the results demonstrate feasibility rather than population wide effectiveness. No power calculation supports generalisation from a cohort of this size, and the six participants were drawn from a single geographic setting with limited variation in age, ethnicity and body composition. The findings should not be extrapolated to the general population. Future work will recruit larger multi-centre cohorts stratified by age, ethnicity, body mass index, medication status and diagnosed diabetic condition, and will include participants with confirmed NIDDM so that the framework can be evaluated against the full range of glycemic states rather than the normoglycemic and prediabetic bands alone.

No large public multimodal NIDDM CGM dataset that pairs glucose with activity, meal and cardiac signals currently exists, so OhioT1DM was selected as the closest publicly available resource. Although the proposed framework demonstrated agreement with HbA1c assessments in six participants, future work will retrain and validate the glucose prediction model on longitudinal NIDDM cohorts. Two specific facets of the gap deserve to be recorded. All twelve OhioT1DM donors were maintained on insulin pump therapy, so the training distribution contains exogenous insulin dynamics that are absent for the experimental subjects, and the OhioT1DM population is likely to exhibit wider glycemic excursions than an undiagnosed NIDDM population, which may bias the model towards over dispersed predictions and therefore towards a conservative coefficient of variation.

The experimental subjects did not wear CGM sensors, so no timestep level ground truth exists against which the 90 days predicted trajectories can be scored. HbA1c is a coarser comparator that summarises roughly two to three months of exposure in a single value and cannot confirm the accuracy of individual excursions. A future study that instruments a subset of participants with reference CGM alongside the smartphone pipeline would allow the predicted trajectories to be validated directly rather than only at the level of the derived risk category.

Meal entries were logged manually, which introduces the well documented risks of portion misestimation, omitted entries and recall bias. Because carbohydrate content is one of the stronger inputs to the glucose module, errors in this channel propagate to the predicted trajectory.

The risk criteria are fixed thresholds adopted from consensus CGM guidance rather than parameters learned from data. This makes the decision transparent and clinically interpretable, but it also means that the framework cannot adapt its boundaries to a specific population, and that a participant who marginally fails a single criterion is treated identically to one who fails all four.

As noted in Section 5.2, the baseline results were produced by the present authors under a matched tuning settings rather than obtained from author released code, since no prior study reports these architectures on a 23 class 1 Hz smartphone corpus.

The two participants flagged as at risk were not followed beyond the study window, so it is not known whether their glycemic profiles progressed, stabilised or reverted. Establishing that the framework identifies individuals who genuinely go on to develop NIDDM requires a prospective design with a follow up period measured in years.

## 6 Conclusion

This novel work presents a GlucoSense architecture based on GATv2 from combining smartphone-based Human Activity Recognition with continuous blood glucose forecasting for early NIDDM risk assessment. The system was configured to use a GATv2-GraphSensor model that was trained using the triaxial accelerometer data collected specifically for this study, and a standalone GATv2 model trained using the OhioT1DM benchmark. The framework captured complex physiological interdependencies governing glycemic behaviour, which cannot be adequately represented by purely sequential architectures, by embedding information about physical activity and heart rate, as well as meal intake, into a graph-structured learning model. Stratification of all participants was achieved during a 90-day evaluation in 6 participants with 3 clinically relevant glucose parameters (mean glucose, TAT, COV). The assigned classifications agreed with subsequent laboratory HbA1c testing for every participant, which offers preliminary support for the internal consistency of the framework within this small cohort. Comparative experiments against sequential, convolutional, attention based and graph based baselines showed that the GATv2 formulation lowered the root mean squared error to 25.60 mg/dL and raised activity recognition accuracy to 98.50%, and every difference remained significant under paired testing after correction for multiple comparisons. GlucoSense is therefore best described as a proof of concept digital health screening system that relies only on a smartphone, a cardiac monitor and the subject’s own dietary reporting, and that could serve as a scalable community level screening framework once it has been validated at larger scale.

Several limitations apply and are set out in full in Section 5.5. In summary, the validation cohort consisted of 6 participants over 90 days, so the results establish feasibility rather than population wide effectiveness, and the statistical generalizability of the risk stratification approach must be confirmed on larger and demographically broader multi-centre cohorts. The blood glucose model was trained on T1DM data because no large public multimodal NIDDM CGM resource exists. The experimental subjects wore no reference CGM sensor, so the predicted trajectories could be compared only against HbA1c at the level of the derived risk category. Manual self-reporting of meal data carries the risks of under or overestimation, omitted entries and recall bias. Going forward, the priorities are to expand the cohort to demographically diverse groups including participants with confirmed NIDDM, to instrument a subset with reference CGM so that trajectory level accuracy can be measured directly, to add further physiological signal modalities, and to investigate subject adaptive modelling strategies that improve generalizability. The glucose predictions would be reinforced by retraining on a matched NIDDM CGM dataset once one becomes available. A prospective follow up extending beyond the observation window would establish whether the individuals flagged as at risk go on to develop NIDDM. An additional opportunity for burden reduction and a decrease in measurement error when tracking meal intake is the use of computer vision to replace manual dietary logging of foods.

## Data Availability

Publicly available datasets were analyzed in this study. This data can be found here: the open source data that support the findings of this study are available at https://webpages.charlotte.edu/rbunescu/data/ohiot1dm/OhioT1DM-dataset.html. The source code is not available due to ongoing development on this research.
